# Proteomic Analysis of Small Extracellular Vesicles From Lymphatic Affluents in Developing Premetastatic Niche in Melanoma

**DOI:** 10.1016/j.mcpro.2025.101472

**Published:** 2025-11-19

**Authors:** Shankar Suman, Liyi Geng, Wendy K. Nevala, Raymond Moore, Chathu Atherton, Xiaowei Zhao, Jaeyun Sung, Ray Guo, James W. Jakub, Richard K. Kandasamy, Sarah A. McLaughlin, Akhilesh Pandey, Svetomir N. Markovic

**Affiliations:** 1 Department of Oncology, Mayo Clinic, Rochester, Minnesota, USA; 2 Department of Quantitative Health Sciences, Mayo Clinic, Rochester, Minnesota, USA; 3 Bioinformatics and Computational Biology Program, University of Minnesota, Rochester, Minnesota, USA; 4 Department of Laboratory Medicine and Pathology, Mayo Clinic, Jacksonville, Florida, USA; 5 Department of Surgery, Mayo Clinic, Jacksonville, Florida, USA; 6 Department of Laboratory Medicine and Pathology, Mayo Clinic, Rochester, Minnesota, USA; 7 Department of Immunology, Mayo Clinic, Rochester, Minnesota, USA; 8 Manipal Academy of Higher Education, Manipal, Karnataka, India

**Keywords:** differentially expressed proteins, lymph nodes, lymphatic fluid, melanoma, premetastatic niche, proteomics, small extracellular vesicles, tandem mass tags

## Abstract

Melanoma is an aggressive form of skin cancer that often metastasizes through lymph nodes (LNs). Lymphatic small extracellular vesicles (sEVs) derived from melanoma play a crucial role in establishing a premetastatic niche (PMN) within the sentinel lymph node (SLN). Therefore, analyzing the proteomic content of tumor-draining lymphatic sEVs that deliver oncogenic signals to the SLN is vital in understanding the PMN. To investigate this, we performed multiplexing (18 samples) using tandem mass tag labeling to profile the lymphatic sEV proteomes obtained from afferent lymphatic channels leading to the SLN of melanoma patients (n = 6), non–cancer-associated afferent lymphatic channels (n = 3), and postoperative lymphatic fluid after LN dissection (n = 9). We identified 595 new proteomic cargoes compared with those reported in ExoCarta and 1003 new cargo proteins relative to three previously reported lymphatic EV datasets. The analysis revealed 145 differentially expressed proteins of melanoma sEVs that link to increased cellular stress and injury pathways and a decrease in extracellular matrix organization (−log[*p* value] >7.0). Analysis of the top 50 differentially expressed proteins included expressions of normal, primary, and metastatic samples across multiple omics datasets. Hierarchical clustering with postoperative samples demonstrated nine upregulated and two downregulated proteins specific to melanoma sEVs, which are associated with melanoma progression (*p* < 0.05). Notably, several common proteins associated with melanoma and postoperative samples were related to the wound healing mechanism. The multiplex immunofluorescence analysis of selected proteins reveals significantly increased expression levels of CD38, galectin-9 (LGALS9), and tenascin-C (TNC) in the lymphatic sinuses of SLN (−) compared with the control LN sinuses. Moreover, higher levels of LGALS9 protein in LN tissue are associated with poor overall survival of melanoma patients (*p* = 0.0018). In summary, this study reveals an altered landscape of sEV proteome in the afferent lymphatic fluid of melanoma, highlighting distinct sEV proteins that are uniquely present in the SLN during PMN development.

## Highlights

• Proteomic analysis of small extracellular vesicles (sEVs) from lymphatic fluid in melanoma patients and control subjects using TMTpro labeling identified key proteins related to premetastatic niche development.
• Melanoma sEV cargoes revealed 145 differentially expressed proteins compared with control lymphatic fluid obtained from the lymphatic afferent channels.
• Multiple omics dataset comparison reveals that melanoma-associated lymphatic sEV cargoes can be involved in metastatic progression.
• Melanoma-negative sentinel lymph node carries more contents of the sEV cargoes, CD38, LGALS9 (galectin-9), and TNC (tenascin-C), in the lymphatic sinuses compared with control lymph nodes.
• LGALS9 was upregulated in melanoma-derived samples, and higher expression is also linked with melanoma prognosis.

## Graphical Abstract

**Figure:**
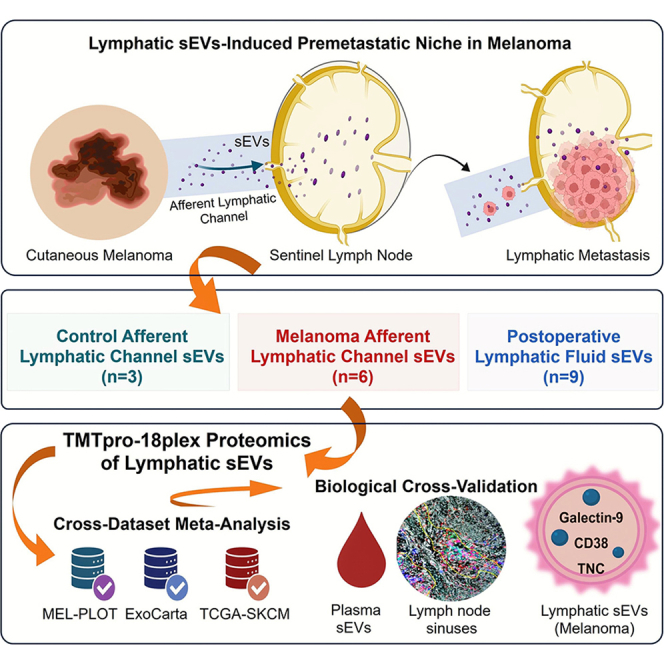

## In Brief

Metastatic melanoma commonly spreads to distant organs *via* the lymphatic system. Melanoma-derived small extracellular vesicles (sEVs) play a crucial role in the development of the premetastatic niche (PMN) in regional lymph nodes. Quantitative proteomic analysis of sEVs from lymphatic fluid in melanoma patients and control subjects identified 145 proteins that may be associated with PMN development through immunological and extracellular matrix organization pathways. High levels of CD38, LGALS9, and TNC were found in the sinuses of the lymph node, supporting potential immunological targets for the PMN in melanoma.

Melanoma is an aggressive form of skin cancer that frequently metastasizes to locoregional lymph nodes (LNs) and distant organs. A critical component of spread involves early metastases to the first tumor-draining LN (sentinel lymph node [SLN]). We have previously described that SLN is immunologically altered before the arrival of the first metastatic melanoma cells (premetastatic niche [PMN]) (1). Melanoma-derived small extracellular vesicles (sEVs) present in the lymphatic fluid draining from the primary tumor (PT) in the skin to the SLN contribute to the establishment of the immunotolerant microenvironment at the SLN, fostering the colonization of melanoma cells (1, 2). Earlier proteomic analysis of SLNs has revealed that many of the modulated proteins in SLNs can be indicative of the pathological state of cancer (3), which can be controlled by sEVs originating from the PT (4). Moreover, a prior proteomic study also revealed that the distinct tissue protein expressions of PTs, LN, and distant metastatic sites are functionally important in relation to melanoma patient survival (5). Several other melanoma tissue proteomic datasets, including Cero and Cuarto datasets, have been integrated at the MEL-PLOT platform, highlighting proteomic relevance to clinical outcomes utilizing PT and LN metastatic samples (6).

Lymphatic sEVs carry a rich source of tissue secretory factors and possess the ability to modulate systemic immune responses through the lymphatic route (7). However, following lymphatic drainage into the systemic circulation, these sEVs are further diluted. Nonetheless, the proteomic cargo derived from plasma EVs has the ability to differentiate patients with melanoma from healthy subjects (8). However, the research focusing on differential EV proteomics within the lymphatic system in the context of melanoma remains limited. During the progression of melanoma, lymphatic sEVs can penetrate the compromised macrophages in the sinuses of the LN, thereby facilitating cancer dissemination *via* lymphatic vessels (9). Furthermore, when endothelial cells receive melanoma sEVs, the expression of VE-Cadherin, epidermal growth factor receptor, and urokinase plasminogen activator receptor is enhanced to promote angiogenesis (10). SEV cargoes have been shown to reprogram stromal fibroblasts to favor PMN development (11). Previous studies also show that sEVs derived from metastatic melanoma cells that propagate to the lung and brain have the capacity to trigger proinflammatory signaling pathways in lung fibroblasts and brain astrocytes, by recruiting immune cells through the expression of inflammation-activating factors, such as Hmgb1, Tslp, and Irf1 (12). Moreover, melanoma sEVs carry immunosuppressive properties as these sEVs directly dampen antitumor CD8+ T-cell function (13).

Gaining a deeper understanding of lymphatic sEVs is crucial for elucidating PMN at the SLN in melanoma. The significance of lymphatic sEVs has been previously recognized as revealing key biomarkers of melanoma and can serve as predictors of disease features. Garcia-Silva *et al*. (14) characterized the exudative seromas in patients with stage III melanoma after lymphadenectomy and established a correlation between the presence of sEV cargoes and the risk of disease relapse. In another study, sEVs of lymphatic exudate and plasma have been analyzed from metastatic melanoma patients after lymphadenectomy, which also reveals the enrichment of tumor-derived factors in sEV within the lymphatic exudate (15). These findings indicate that metastatic melanoma patients may exhibit higher levels of secreted tumor factors in lymphatic fluid compared with blood plasma across various stages of melanoma progression. While several studies have probed blood-based comparative studies on EV proteomes across multiple cancers, including melanoma, there remains a noticeable gap in research studying the differences of sEV in the lymph of noncancer and cancer patients (16, 17). Unlike blood, lymphatic fluid circulates through a unidirectional network of vessels that originates from interstitial fluid in peripheral tissues, and a continuous influx of afferent lymph is critical for maintaining LN physiological function (18). Furthermore, afferent lymph also carries tissue-specific signatures and plays a critical role in evaluating the tissue-specific antigenic and inflammatory burden (19). To gain a comprehensive understanding of the changes in the LN induced by lymphatic sEV in melanoma, we conducted a proteomic analysis of sEVs collected from the lymphatic fluid of the intraoperative afferent channel. Our study compares critical lymphatic sEV in the afferent channel lymphatic fluid, focusing on the changes in proteome landscape between control and melanoma cases. It is important to recognize that LN acts as a site of "dynamic inflammation," constantly undergoing changes in response to foreign threats. This process involves localized swelling and activation of immune cells, followed by a return to its normal size once the threat has been neutralized (20). Therefore, we also utilized postoperative lymphatic drainage to compare with altered proteins of the afferent channel proteome in melanoma. All lymphatic sEV protein samples were labeled with isobaric tandem mass tags for quantitative proteomics, which helps identify and quantify proteins within the lymphatic sEV samples. Our approach aims to identify the melanoma sEV cargoes that deliver oncogenic signals to SLNs, providing insights into how melanoma lymphatic sEVs modify SLN for the development of a PMN.

## Experimental Procedures

### Informed Consent, Patient Details, and Sample Collection

We prospectively collected lymphatic fluid from afferent lymphatic channels leading from the primary melanoma to the SLN as described earlier (21). Control lymphatic fluid was obtained from the afferent lymphatic channels of patients who underwent noncancer surgeries, specifically prophylactic mastectomy, and who exhibited no evidence of malignancy. These samples were collected by following the Declaration of Helsinki and approval by the Mayo Clinic Institutional Review Board (IRB: 10-000806). Lymphatic tracking using blue dye is a general clinical practice in melanoma operations to identify SLN, which is the LN most likely to harbor metastasis. Using this method, lymphatic channels leading from the primary cutaneous melanoma to the LN were collected during surgery. The lymphatic channels were clipped at both ends to prevent lymph leakage before being submitted to the laboratory. For the control samples, the blue dye was injected into the breast in a standard subareolar/periareolar fashion to detect the blue afferent lymphatic channel draining into the axilla. These controls were selected from patients who were undergoing prophylactic mastectomies. Lymphatic fluid was also collected from postoperative lymphatic fluid after lymphadenectomy drains under an approved clinical protocol (IRB# 08-004581). Lymphatic fluid from postoperative lymphadenectomy drains served as another control to filter out nonmelanoma alteration in lymph that may be related to the procedure of surgical resection and the wound healing process. To validate some of our proteomics findings, we also utilized four LN tissue samples: two from normal LN and two from melanoma patient SLN (−), along with blood plasma samples from six melanoma patients and six healthy donors. The clinicopathological details of all biospecimens used in the study are available in Supplemental Data, which include clinicopathological details of lymphatic fluid samples (Supplemental Table S1), the details of LN tissues analyzed using multiplex immunofluorescence (MxIF) (Supplemental Table S2), and the details of blood plasma samples (Supplemental Table S3). Informed consent was obtained from each participant for the prospective collection of biospecimens.

### Separation of sEVs

Upon receipt of the freshly resected lymphatic afferent channel, surgical clips from both ends of the channels were carefully removed, and the lymphatic fluid within the channel was subsequently perfused with RPMI media using a 30-gauge needle using a dissecting microscope. These media were further filtered through a 0.8-μm syringe filter prior to the isolation of sEVs. To remove the debris, lymphatic fluid was centrifuged at 500*g* for 10 min, followed by a second centrifugation at 2000*g* for 20 min. From each sample, 1 ml was processed using a qEV1 70 nm column (IZON) (Malvern). The column was equilibrated and eluted with 1x PBS buffer according to the manufacturer's instructions. Each fraction consisting of 0.7 ml was collected per fraction, and the first six fractions were pooled (∼4 ml) for the subsequent analysis. The lymphatic sEVs were concentrated by using a 30 kDa cellulose spin column (4 ml) at 3000*g* for 15 min to remove excess fluid. The sEVs were then resuspended in 150 μl of 1x PBS. The column was washed with 50 μl of 1% CHAPS in 1x PBS, and both the wash and the resuspended EVs were mixed to a final volume of 200 μl. The resulting concentrated samples were used for further downstream analysis, including transmission electron microscopy, immunoblotting, nanoparticle tracking analysis (NTA), and proteomic analyses. miRCURY Exosome Serum/Plasma Kit (catalog no.: 76603; Qiagen) was used to collect sEV human blood plasma of healthy and melanoma subjects by following the manufacturer's protocols.

### Nanoparticle Tracking Analysis of Lymphatic sEVs

The particle size distribution and concentration of sEV particles were analyzed using the NanoSight NS300 with NTA 3.4 software. sEV samples were diluted 200-fold in 1x PBS. Measurements were performed at a camera level of 14 and a syringe pump speed of 60. Particle tracking analysis was conducted with a detection threshold of 5. The sEV particle concentration was reported per milliliter of sample after being measured in triplicate.

### Transmission Electron Microscopy

These vesicles were adhered to carbon-coated 200 mesh copper grids. The grids were subsequently washed in 0.1 M phosphate buffer at pH 7.0 and fixed with a solution containing 4% paraformaldehyde and 1% glutaraldehyde. Finally, they were negatively stained with 1% phosphotungstic acid. Micrographs were further acquired using a JEOL1400 Plus transmission electron microscope (JEOL).

### Western Blotting

The sEV protein lysates were first prepared with radioimmunoprecipitation assay buffer supplemented with protease inhibitor cocktail, and protein concentration was measured. Equal protein concentration of samples was loaded onto a 4% to 15% Criterion TGX Precast Midi Protein Gel (catalog no.: 5671083; Bio-Rad) and separated using a Bio-Rad electrophoresis unit with 1x Tris–glycine–SDS running buffer (catalog no.: 1610732; Bio-Rad). The proteins on the gel were further transferred to polyvinylidene fluoride membrane using Wet/Tank Blotting Systems (Bio-Rad), which was confirmed by Ponceau S staining. Before probing the primary antibodies against sEVs, tetraspanins, and other proteins, polyvinylidene fluoride membranes were blocked with 5% nonfat milk in a 1× Tris-buffered saline with Tween-20 (TBST) solution. We used primary antibodies against CD9 (ab263019; abcam), ALIX (ab117600; abcam), syntenin-1 (27964; Cell Signaling Technology), albumin (catalog no.: 4929; Cell Signaling Technology), calnexin (ab22595; abcam), galectin-9 (LGALS9, AF2045; R&D Systems), CD3D (ab229280; abcam), and CD38 (ab226034; abcam) in blocking buffer under constant shaking for overnight at 4 °C. The membranes were washed with 1X TBST (3x), and horseradish peroxidase–labeled secondary antibodies against the host primary antibodies were added for 2 h at room temperature. Chemiluminescent substrates (Bio-Rad) were added to the membrane after removing unbound antibodies with 1x TBST (3x), and blots were developed on X-ray films and further scanned and analyzed by ImageJ software (https://imagej.net/ij/) (22), and relative fold changes were determined after total protein normalization.

### TMTpro Labeling and Mass Spectrometry Analysis

#### Sample Preparation and TMTpro Labeling

Isolated sEV samples were processed using the S-Trap micro cartridges (ProtiFi.com) following the recommended protocol. Briefly, the samples were lyophilized and solubilized in 23 μl of 5% SDS–50 mM triethylamine bicarbonate (TEAB; pH 8.5), reduced with 5 mM Tris(2-carboxyethyl)phosphine–50 mM TEAB (pH 8.5) at 50 °C for 15 min, and alkylated with 10 mM iodoacetamide/50 mM TEAB (pH 8.5) for 20 min at room temperature before acidification with 2.5% phosphoric acid to create the protein suspension for loading into the S-trap cartridge. After several washes with 100 mM TEAB–90% MeOH, 20 μl of (0.05 μg/μl) Worthington trypsin/50 mM TEAB (pH 8.5) was added and incubated overnight at 37 °C. The peptides are eluted with 0.2% formic acid in water and 0.1% TFA–50% acetonitrile–50% water and lyophilized. The peptides were solubilized in 100 μl 300 mM Hepes (pH 8.5) buffer and labeled with 500 μg TMTpro 18-plex reagents in 20 μl acetonitrile for 1 h at room temperature and quenched with 5% hydroxylamine. A 5 μl aliquot from each labeling reaction was combined and analyzed by nanoLC-tandem mass spectrometry (MS) to confirm that the percentage of TMTpro-labeled peptide-spectrum matches (PSMs) relative to total PSMs exceeded 99%. The remainder of the reactions was pooled by normalizing with the reporter ion intensities to combine equal amounts of each sample. Excess TMTpro reagent was removed from the mixture using a Waters Sep-Pak C18 Plus long cartridge. The cartridge was loaded with 0.1% TFA–5% acetonitrile and washed with 0.1% TFA–60% acetonitrile to elute the peptides, which were then lyophilized.

#### Peptide Fractionation With Basic Buffer HPLC

The dried TMTpro peptide mix was solubilized in 5% dimethyl sulfoxide–5 mM ammonium formate (pH 8.5) and fractionated using basic pH reverse-phase HPLC on a Thermo Ultimate 3000 RSLC HPLC system with a Waters XBridge peptide BEH C18, 3.5 mm, 4.6 mm column. The mobile phases were aqueous 5 mM ammonium formate, pH 8.5 for solvent A and 5 mM ammonium formate, pH 8.5–90% acetonitrile–10% water for solvent B. A flow rate of 0.5 ml/min was used, and gradient conditions were 5%B to 60%B over 60 min, to 80%B in 2 min and held for 5 min. A total of 96 fractions were collected over the 80-min method and were concatenated to 12 fractions and lyophilized prior to nanoLC-tandem MS analysis.

#### NanoLC-Tandem MS Data Acquisition for Total Protein

The peptide fractions were analyzed by nanoLC-tandem MS using a Thermo Scientific Orbitrap Eclipse mass spectrometer coupled to a Thermo Vanquish Neo UHPLC system with 0.1% formic acid in 98% water–2% acetonitrile for solvent A and 0.1% formic acid in 80% acetonitrile–10% isopropanol–10% water for solvent B. Each fraction was solubilized in 0.1% formic acid and pumped onto a Halo C18 2.7 μm EXP stem trap (Optimize Technologies) with solvent A at a flow rate of 8 μl/min. The trap was placed in line with a Bruker PepSep C18 2.7 μm, 40 cm × 100 μm column, and the peptides were separated at a flow rate of 350 nl/min with a gradient of 4%B to 40%B over 120 min, then 40%B to 90%B over 12 min, with a 5-min hold at 90%B for 5 min. Data were acquired using a real-time search MS3 method for TMTpro quantitation. MS1 scans were performed in the Orbitrap with a mass range of 350 to 1500 *m/z*, automatic gain control at 100%, 50 ms maximum injection time, and resolution at 120,000 at 200 *m/z*. Ions were selected with an isolation width of 0.7 and fragmented by collision-induced dissociation in the ion trap using the turbo scan rate, and the MS2 spectra were searched in real time against a reviewed UniProt human database with parameters set to allow 1 missed cleavage, static modifications of carbamidomethyl cysteine, TMTpro-labeled lysine, TMTpro-labeled peptide N terminus, and oxidized methionine as variable modifications. Peptide spectral matches meeting minimum values of 1.2 for Xcorr, 0.1 for dCN, and a delta mass of 12 ppm or less triggered an MS3 scan event for TMTpro reporter ion quantitation. Up to 10 fragment ions in the mass range 200 to 1500 from the matched spectra were isolated by synchronous precursor selection to generate the reporter ion MS3 spectra with higher collision dissociation fragmentation at 55% normalized collision energy and Orbitrap scanning from 100 to 500 *m/z* at 50 k resolution. The MS3 automatic gain control setting was 500% with the maximum ion injection time of 86 ms. Dynamic exclusion was used to prevent ions selected for MS2 and any ions within an *m/z* of 7 ppm from being selected for fragmentation for 25 s. The close-out feature was implemented to restrict the MS3 trigger to a maximum of six peptide matches to a protein in each fraction run. The MS proteomics data have been deposited to the ProteomeXchange Consortium *via* the PRIDE (23) partner repository with the dataset identifier PXD063898 and 10.6019/PXD063898".

#### Protein Identification and Quantitation

The MS raw data files were analyzed using Proteome Discoverer 3.0 (Thermo Scientific) and set up for MS3 reporter ion quantification with 18-plex TMTpro isobaric labels. The raw files were searched against a reviewed UniProt human downloaded June 2024 database (20,349 entries) using Sequest HT with parameters set for full trypsin specificity, allowing two missed cleavages and the variable modifications, oxidized Met and N-terminal protein acetylation. Fixed modifications were carbamidomethyl cysteine, TMTpro lysine, and peptide N-terminal TMTpro. Mass tolerances were set at 10 ppm for precursor ions and 0.5 Da for MS2 fragment ions. Protein identifications were filtered at 1% false discovery rate (FDR) using the Percolator node. The MS3 TMTpro reporter ion channel intensities were reported with correction factors applied to PSMs and no filtering with isolation interference set at 100%. The matched proteins and raw reporter ion intensity values were exported to Excel, and group comparisons were made with an in-house R script.

### Statistical Analysis of LC–MS/MS Data

To facilitate downstream analyses, reporter ion intensities from TMTpro-based proteomics data were preprocessed by removing proteins with missing values across samples, followed by sample-wise normalization, imputation of missing values, and log2 transformation. First, proteins with missing intensity values in more than 25% of the total samples were excluded. Next, sample-wise median centering was performed to normalize intensity values across samples. Specifically, each TMTpro channel represents one sample. For each channel, the median of all nonzero reporter ion intensities was calculated, and every intensity value within that channel was divided by this median to adjust for loading and labeling differences. Then, missing intensity values were imputed using half of the global minimum nonzero intensity value, which was defined as the smallest nonzero reporter ion intensity observed across all proteins and samples. Finally, all intensity values were log_2_ transformed. Statistical differences between groups were assessed using a two-sample *t* test assuming unequal variances (Welch’s *t* test), and differentially abundant proteins were identified based on |log_2_[mean fold change]| ≥1.0 and *p* value <0.05. Furthermore, volcano plots and other graphical representations were created using GraphPad Prism, version 10, software (GraphPad Software, Inc).

### Bioinformatics and Data Visualization

Lymphatic sEV proteomes were compared with reported human sEV proteomic cargoes (release data: March 20, 2025) from the ExoCarta database (exocarta.org), and their comparison was made with Venn diagram analyses (VENNY 2.1). We compared our sEV proteomics data with previously reported lymphatic datasets by Garcia-Silva *et al.* (14), Broggi *et al.* (15), and Nanaware *et al*. (19). Moreover, we compared our proteomic data against those from studies of Nevus-associated melanoma (NAM) *versus* corresponding nevi (24) to evaluate our top differentially expressed proteins (DEPs). In addition, we compared the proteomic data derived from melanoma cell lines within the NCI-60 EVs (25), also against control melanocytes *versus* melanoma (14, 26), focusing on upregulated proteins in our study. To gain insights into our modulated data, we employed Ingenuity Pathway Analysis (IPA; Qiagen) to analyze canonical pathways with a significance threshold of -log(*p* value) >7.0, as well as to assess the network and cellular localization of all melanoma-modulated proteins. In addition, we conducted Gene Ontology analysis, Kyoto Encyclopedia of Genes and Genomes analysis, and other pathway enrichment analyses for the functional annotation of the proteomic data using the DAVID (database for annotation, visualization, and integrated discovery) and ShinyGO 0.82 web tools. Furthermore, we utilized the STRING (search tool for the retrieval of interacting genes/proteins), version 12.0, database (https://string-db.org/) to investigate protein–protein interaction networks, especially Markov Cluster algorithm clustering method and perform functional enrichment analysis of the upregulated sEV cargo proteins in melanoma. We also used the TNMplotter (27) to analyze gene expression, gene correlation (Spearman's correlation), and signature expression analyses for the DEPs that use RNA-Seq data from The Cancer Genome Atlas (TCGA)-Skin Cutaneous Melanoma (SKCM) datasets. Tumor and metastatic data of the SKCM dataset were analyzed using the TIMER3.0 webserver (28). The analysis of gene expression among normal, tumorous, and metastatic tissues was conducted using the Kruskal–Wallis test. Gene signature analysis was performed by TNMplotter, which calculates the average of the selected gene signature for each patient individually based on RNA-Seq data (https://tnmplot.com/analysis/). The proteomic data of PTs and LN metastatic tumors were obtained from the Cero and Cuarto datasets of MEL-PLOT (www.tnmplot.com/melanoma) (6). For the survival plot analysis of proteins, the Cero proteomic dataset with LN tumors was utilized.

### Selected Proteomic Cargo Analysis of sEVs in Lymphatic Sinuses

The tissue sectioning and H&E staining of normal LN as well as melanoma uninvolved SLNs or SLN (−) were conducted by the Pathology Research Core at the Mayo Clinic in Rochester, Minnesota. Sequential tissue sections were collected for MxIF analysis of sEV proteomic cargoes (tenascin-C [TNC], CD38, and LGALS9) along with cellular classification markers. Briefly, formalin-fixed, paraffin-embedded sections (5 μm) were prepared by baking slides at 70 °C for 1 h, followed by deparaffinization in xylene and rehydration through graded ethanol. Permeabilization was done using 0.3% Triton X-100, and antigen retrieval employed citrate buffer (pH 6) and Tris–EDTA buffer (pH 9.5). After blocking with 10% donkey serum and 3% bovine serum albumin, slides were counterstained with 4′,6-diamidino-2-phenylindole and mounted with a nonhardening antifade medium. Primary antibodies were purified *via* HiTrap Protein A or G columns and conjugated with NHS–ester fluorophores using anhydrous dimethyl sulfoxide. They were stabilized in 1x PBS containing 0.2% bovine serum albumin and 0.09% sodium azide at a concentration of 300 μg/ml. MxIF imaging utilized staining of antibodies at 10 μg/ml. After staining, slides were washed in PBS, cover-slipped, and imaged using the Cell Dive Imager. Whole-slide images were captured at 10× magnification, and regions of interest in the LN sinus were imaged at 20× magnification. Each field of view (FOV) was systematically examined and annotated for LN sinuses utilizing QuPath software. This process involved the selection of regions of interest characterized by proper H&E features with a corresponding high density of macrophages, employing CD163 and CD68 as markers, adhering to previously published methods (29, 30) and was executed by an experienced board-certified surgical pathologist (R.G.). The mean fluorescence intensity for each annotated sinus area of the FOVs was calculated and analyzed in both control LN and SLN (−) groups.

### Experimental Design and Statistical Rationale

In our study, we focused on three distinct groups of samples for a thorough comparative TMTpro proteomics analysis, which include afferent lymphatic sEVs obtained from SLNs of patients with melanoma; afferent lymphatic sEVs from control LN from noncancer subjects, and lymphatic sEVs from postoperative lymphatic fluid after lymphadenectomy drains. Samples included in the proteomics experiment were the afferent lymphatic channel of melanoma (n = 6) and control (n = 3) as well as postoperative lymphatic fluid control (n = 9). This analysis was done to evaluate the differential proteomic cargoes in melanoma-derived sEVs in the lymphatics reaching at LN and how these altered cargoes in melanoma-derived sEVs involve lymphatic inflammatory or wound healing mechanisms. Our proteomic data were compared with proteins from ExoCarta (sEV database), previously published datasets on lymphatic EV. We focused on the top 50 significant DEPs to evaluate metastatic characteristics of these proteins using melanoma primary and metastatic tissues at transcriptomic (TCGA-SKCM) and proteomics levels. Furthermore, we analyzed some selected key proteins for their differential expression in the lymphatic sinuses, as these are the pathways for lymphatic sEV delivery to the SLN. Moreover, expression of cargo proteins was also validated in the blood plasma sEVs, collected from the healthy subjects (n = 6) and melanoma patients (n = 6). Two-tailed Student's *t* tests were employed to assess the differences in key modulated proteins between the control and melanoma samples as well as between tissue samples (PT and LN) derived from the Cuarto and Cero datasets of melanoma, which can help to identify the sEV cargoes with LN metastatic behavior. The Cuarto dataset consists of LN metastases (n = 11) and PTs (n = 16), whereas the Cero dataset includes LN metastases (n = 20) and PTs (n = 52).

## Results

### Isolation and Characterization of Lymphatic sEVs Obtained From Surgically Dissected Lymphatic Channels

**Fig. 1:**
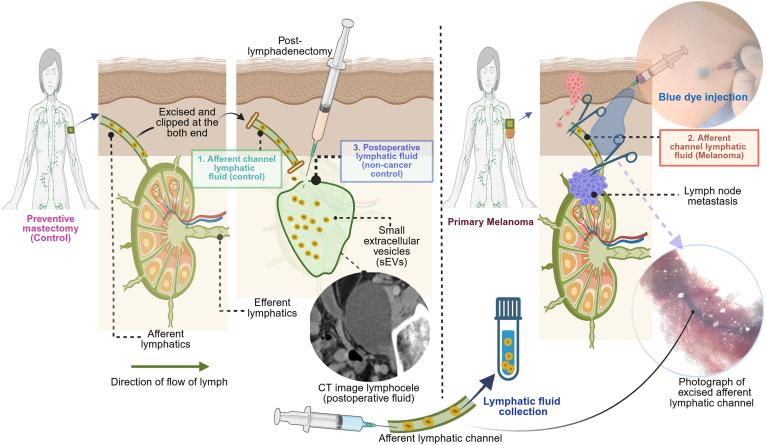
**Collection of lymphatic fluid for sEV isolation from control cases (preventive mastectomy) and cutaneous melanoma cases.** The afferent lymphatic channel that drains lymph from peripheral tissue to the sentinel lymph node (LN) was identified by surgeons using blue dye injection as part of their routine procedure. The excised intraoperative afferent lymphatic channel was clipped, and lymphatic fluid was collected by washing the channel with syringes from the following subjects: 1. Individuals undergoing preventive mastectomy (control). 2. Melanoma patients undergoing lymphadenectomy for LN metastasis screening. In addition, lymphatic fluid that drains postlymphadenectomy is referred to as postoperative lymph fluid and categorized as 3. postoperative lymph. These three categories of samples used isolation of lymphatic sEVs, and the proteomic analysis was performed. sEV, small extracellular vesicle.

**Fig. 2:**
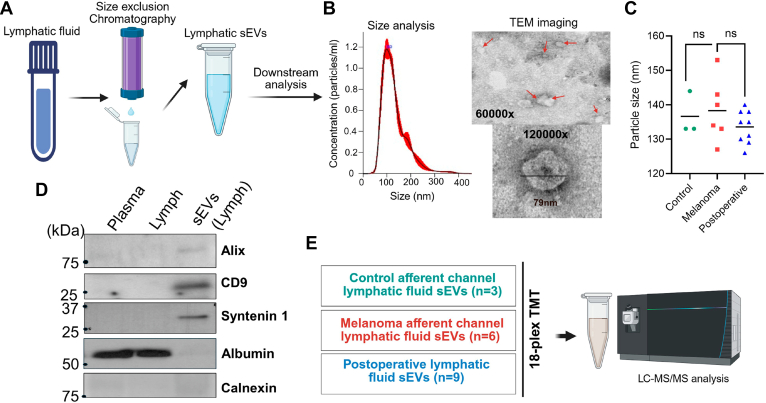
**Experimental workflow for characterization of lymphatic sEVs and proteomic analysis.***A*, lymphatic fluid was used to isolate sEV particles through size-exclusion chromatography (qEV1 70 nm column; IZON), followed by concentration using a cutoff column (30 kDa). The sEVs were characterized using nanoparticle tracking analysis (NTA) performed in triplicate. *B*, transmission electron microscopy (TEM) analysis was conducted to examine the size and morphology of lymphatic sEV. *C*, a *dot plot* indicates the average size of sEV from each sample in control, melanoma and postoperative lymphatic fluid. *D*, *Western blot* analysis was performed to assess the presence of classical sEV markers, including Alix, CD9, and syntenin-1, as well as negative makers, albumin and calnexin in plasma, lymph, and exosomal particles. *E,* the TMTpro workflow involved labeling lymphatic sEVs collected from afferent channel control, melanoma, and postoperative samples with 18 different TMTpro tags, which were further pooled, cleaned, and then analyzed using mass spectrometry. sEV, small extracellular vesicle; TMT, tandem mass tag.

We established the collection of lymphatic fluids from the afferent lymphatic channels from control and melanoma cases, as shown in the method (Fig. 1). Our study delineated the characterization of lymphatic fluid–derived sEVs and compared them with the source lymphatic fluid (Fig. 2). Lymphatic sEVs were isolated from three different sources, including the afferent lymphatic channel of melanoma, the afferent lymphatic channel of control (preventive mastectomy), and postoperative lymphatic fluid (collected postlymphadenectomy from breast surgery). Although the concentration of sEV particles varied between samples, there were no significant differences in average particle sizes among the three groups (Fig. 2). The violin plots of sEVs provide similar size profiles across all samples (Supplemental Fig. S1). Moreover, transmission electron microscopy analysis validates the NTA results, and we observed the sEV particle size within 200 nM, which is a key feature of sEV as recommended by Minimal Information for Studies of Extracellular Vesicles (31). We performed Western blot to verify tetraspanins (CD9, CD63), ALIX (PDCD6IP), considered markers of sEVs, and we also checked albumin (abundantly present in the plasma and lymph) and calnexin (an endoplasmic reticulum protein, which serves as a negative marker of sEVs) (Fig. 2*D*). The absence of albumin and calnexin in lymphatic sEVs suggests no contamination from blood, lymph, or cellular sources.

### Proteome Analysis of Lymphatic sEVs Identifies Unique Set of Proteins

**Fig. 3:**
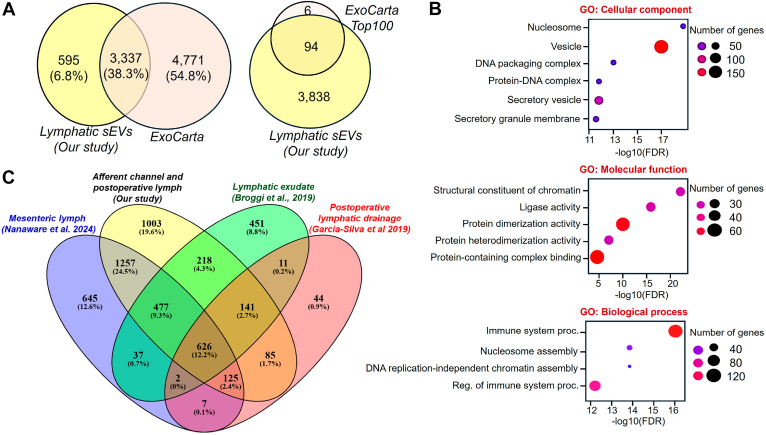
**Lymphatic sEVs exhibit distinct proteomic cargo profiles that differ from those reported in datasets.***A*, Venn diagram displays the unique cargoes found in lymphatic sEVs in comparison to the total proteins of human ExoCarta as well as ExoCarta top 100. *B*, Gene Ontology (GO) analysis of the unique proteins identified in lymphatic sEVs. *C*, Venn diagram analysis of our dataset with three published lymphatic datasets. sEV, small extracellular vesicle.

The dynamic nature of LNs is largely influenced by lymphatic fluid, which comes from the peripheral tissue and is highly controlled as it enters the LN. Lymphatic sEVs are unique in carrying cargo from the peripheral tissue to regulate LN physiological function. Lymphatic sEVs are also considered efficient in delivering signals to the target organ by delivery into the systemic circulation, preparing LNs for forthcoming threats. We analyzed the sEV proteome from the lymphatic fluid from the surgically removed axillary lymph afferent channels as well as the postoperative lymphatic fluid. The sEVs from the lymph identified 3932 proteomic cargoes as identified using a stringent cutoff of the FDR (<0.01) (Supplemental File S1). Comparing the lymphatic sEV proteome to all available cargoes of human sEVs available at ExoCarta, we identified 595 unique proteins in lymphatic sEVs (Fig. 3*A* and Supplemental File S2). Among these, 94 proteins of lymphatic sEVs matched the top 100 proteins found in ExoCarta (Fig. 3*A*). Gene Ontology analysis of these unique proteins showed enrichment for proteins associated with vesicles, immune system processing activity, and chromatin assembly (Fig. 3*B*). Furthermore, most of these proteins are connected to protein families, including transporter, enzyme, receptors, etc (Supplemental File S2). This highlights the significance of lymphatic sEVs in the regulation of LN functions. We also compared the proteins with three previously reported proteomic datasets, among which two used lymphatic exudates collected as postoperatively lymph, and one study was based on the mesenteric afferent lymph (14, 15, 19). This comparative analysis provided 1003 (19.6%) unique proteins in our proteomic dataset, along with 626 (12.2%) common proteins found in all datasets (Fig. 3*C* and Supplemental File S3). The five most enriched unique proteins were IGLC2, MYL12B, CALM3, TUBA1A, and HSPA1B, with coverage over 60%. Among all common 626 proteins, 70 proteins were among top 100 ExoCarta proteins, which fall under well-reported sEV markers like tetraspanin (CD9, CD81), endosomal sorting complex required for transport proteins (ALIX or PDCD6IP), trafficking proteins (RAB14, RAB5C, and RAB7A), integrins (ITGA6, ITGB1), and syntenin-1 (SDCBP). Furthermore, Western blot analysis confirmed the presence of proteins like ALIX, CD9, and syntenin-1 in the lymphatic sEVs (Fig. 2*D*).

### Altered Proteomic Map of sEVs From Afferent Lymphatic Channel Lymphatic Fluid of Melanoma and Noncancerous Lymphatic Channel Control

**Fig. 4:**
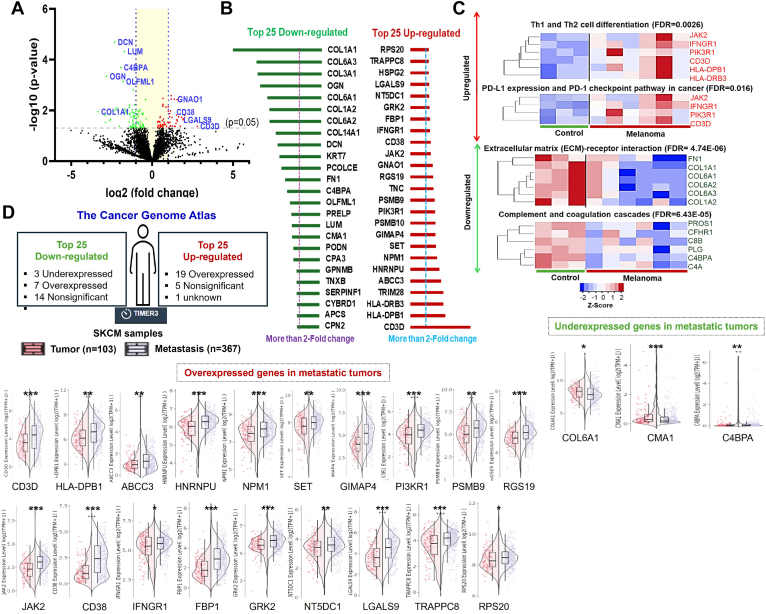
**Melanoma lymphatic sEVs contain unique proteomic cargoes to establish oncogenic pathways in the SLN.***A*, the volcano plot illustrates the significant upregulation and downregulation of proteins in the sEVs derived from the afferent lymphatic channel of melanoma in comparison to the control group. *B*, *top* 25 upregulated and *top* 25 downregulated proteins in melanoma lymphatic sEVs compared with control. *C*, KEGG pathway enrichment analysis reveals that modulated proteomic cargoes are associated with immunoregulation and cellular structural remodeling. *D*, comparison of *top* 50 modulated proteins with SKCM datasets in primary (n = 103) and metastatic tumors (n = 367). Significance levels are indicated as follows: ∗*p* < 0.05; ∗∗*p* < 0.01; and ∗∗∗*p* < 0.001. KEGG, Kyoto Encyclopedia of Genes and Genomes; sEV, small extracellular vesicle; SKCM, skin cutaneous melanoma; SLN, sentinel lymph node.

Our studies analyze the proteome of lymphatic sEVs of melanoma *versus* control samples from the afferent lymphatic channels. In this setting, we identified 145 significant DEPs between control and melanoma lymphatic sEVs (*p* ≤ 0.05) (Supplemental File S4). Among them, 28 proteins were upregulated (≥2-fold change) and 53 proteins were downregulated (≥2-fold change) in the lymphatic sEVs of melanoma compared with control subjects (Fig. 4*A*). The top 25 upregulated and top 25 downregulated proteins are shown in Figure 4*B*. The IPA of DEPs shows their link to increased cellular stress and injury pathways and a decrease in extracellular matrix (ECM) organization (−log[*p* value] >7.0) (Supplemental Fig. S2) and associates with the activation of STAT1-, TNF-, and IRF7-mediated pathways (Supplemental Fig. S3). Moreover 99 proteins were matched with melanoma-related pathway (Supplemental Fig. S4). Gene Ontology analysis revealed that differences in enriched proteins related to cellular components and post-translational modifications among the upregulated and downregulated protein cargoes in melanoma. Most of the downregulated proteins (34/53) were secreted proteins (FDR = 3.08 × 10^−17^), and 41/53 were glycoproteins (FDR = 1.54 × 10^−09^) in nature (Supplemental Table S5). Moreover, pathway analyses demonstrate that most upregulated proteins are associated with the enrichment of PD-L1 expression, and the PD-1 checkpoint pathway, as well as the Th1 and Th2 cell differentiation pathways (FDR <1 × 10^−2^) (Fig. 4*C*). Notably, these pathways were primarily related to immune regulation during PMN formation (32). In contrast, the proteins that were downregulated are linked to pathways involved in ECM–receptor interactions, and the complement and coagulation cascades (FDR <1 × 10^−4^) (Fig. 4*C*), all of which are highly relevant to the remodeling of the SLN (33). Decreased levels of complement and regulatory proteins in lymphatic sEVs, such as PROS1, CFHR1, C8B, PLG, C4BPA, and C4A, may weaken local immune surveillance and disrupt complement-mediated clearance (34). For example, PROS1 in plasma EVs supports anti-inflammatory signaling in macrophages (35); CFHR1 and C4BPA are considered fluid-phase regulators of complement pathways (36). PLG participates in fibrinolysis (37), and C8B helps in the clearance of cancer cells (38). These findings show that downregulation of these proteins in sEV may suppress complement activity, impair apoptotic cell removal, and create an immunosuppressive and proteolytic microenvironment toward the development of a PMN in SLN.

### Altered Lymphatic sEV Proteins Identified as Significant Correlates of Samples Obtained From Metastatic Sites

**Fig. 5:**
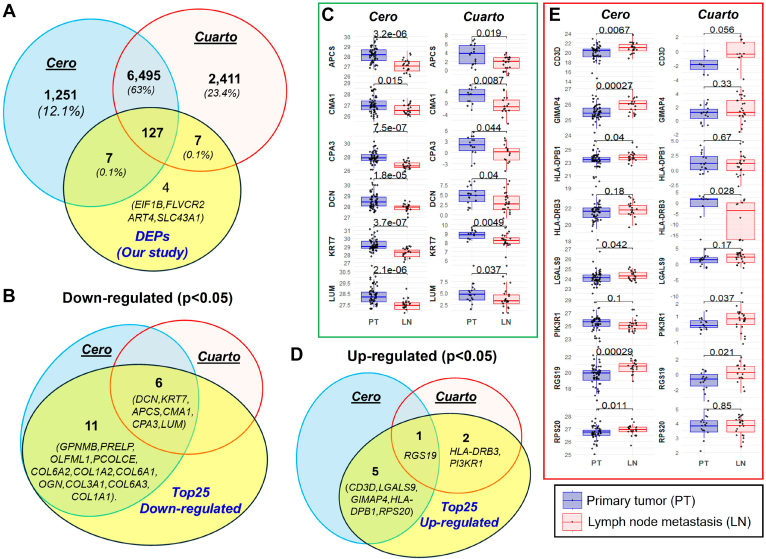
**Comparative analysis of sEV deregulated proteins with melanoma Cero and Cuarto proteomic datasets.***A,* Venn diagram analysis of all differentially expressed proteins (DEPs) in melanoma sEVs with Cero and Cuarto datasets. The top 25 DEPs, included both upregulated and downregulated proteins were compared across both these datasets by Venn diagram, and their expression level are shown by box plots. *B–C,* among the top 25 downregulated DEPs, six common proteins are shown using box plots. *D–E,* among the top 25 DEPs, with eight upregulated proteins from either the Cero or Cuarto dataset displayed through box plots. sEV, small extracellular vesicle.

To investigate the association of these proteins with metastatic progression, we compared the top 25 DEPs with the TCGA-SKCM datasets. Interestingly, 19 of 25 upregulated proteins are overexpressed at the transcriptomic level in the metastatic melanoma tissue compared with PT; however, 3 of 25 top downregulated proteins are associated with metastasis compared with PTs (Fig. 4*D*, and Supplemental Figs. S6 and S7). We further analyzed the expression of these proteins to see if these DEPs are associated with LN metastatic tumors compared with PTs, using the Cero and Cuarto datasets of MEL-PLOT (6). Our findings revealed that 141 of 145 DEPs were previously identified in either Cero or Cuarto datasets (Fig. 5*A*). We also found that 17 of the top 25 downregulated proteins and 8 of the top 25 upregulated proteins matched their expression pattern in the LN tumor compared with the PT (Fig. 5, *B*–*E* and Supplemental Fig. S8). Furthermore, in our multiple comparative evaluations, proteins such as CMA1 and COL6A1 displayed downregulated, whereas proteins, including CD3D, HLA-DPB1, LGALS9, GIMAP4, RPS20, and RGS19, demonstrated upregulation at both the transcriptomic and proteomic levels in metastatic samples (Figs. 4*D* and 5). While comparing our DEPs in nevi with those in corresponding NAMs, which serve as indicators of melanoma progression from premalignant lesions, we identified that TNC was upregulated in 1 of 16 matched top 25 DEPs. Furthermore, 22 of the top 25 proteins examined demonstrated downregulation in NAMs relative to nevi, with statistical significance (*p* < 0.05) as illustrated in Supplemental Figure S9. This finding suggests that the decreased levels of melanoma lymphatic sEV cargo may be directly correlated with the primary site of melanoma. We also investigated if upregulated proteins in lymphatic sEV were found in the melanoma cell line–derived EVs. Our analysis revealed that 57 of 62 upregulated DEPs were identified in the NCI-60 melanoma EVs (Supplemental Fig. S10, *A* and *B*). In comparing all DEPs with datasets provided by Garcia-Silva *et al*., we noted several proteins that were modulated in EVs from melanoma cell lines *versus* primary melanocytes (Supplemental Fig. S10*C*). Moreover, TNC was found to be elevated in the EVs from melanoma cells compared with primary melanocytes in key previously published studies (Supplemental Fig. S10*D*).

### Comparative Proteomics Revealed Proinflammatory and Wound Healing Factors in Melanoma Lymphatic sEVs

**Fig. 6:**
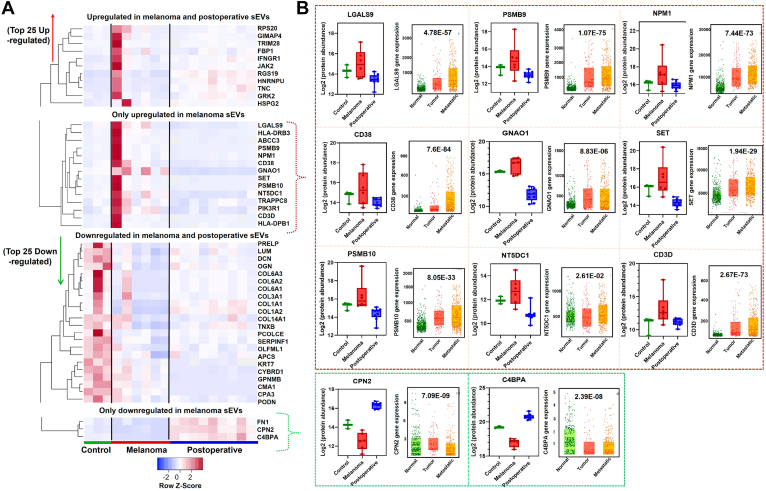
**Proteomic analysis of control, melanoma, and postoperative lymphatic fluid sEVs demonstrates distinct proteomic cargoes of melanoma.***A*, heatmap indicating sEV proteomic differences among control, melanoma, and postoperative lymphatic fluid. *B*, box plot with individual data points display proteins of melanoma and postoperative lymphatic fluid, compared with the control, along with expression plots from TNMplotter, indicating the direct involvement of the highlighted proteins in the melanoma metastasis. This analysis utilizes RNA-Seq data derived from 474 samples of normal tissues from noncancer patients, 103 tumor samples, and 368 metastatic cases. These findings underscore the significance of unique proteomic cargoes within the context of melanoma. sEV, small extracellular vesicle.

We observed a significant alteration in the proteome of sEVs collected from the postoperative lymphatic fluid in comparison to the control afferent channel (Supplemental Fig. S11). One key reason for these changes is the presence of high tissue factors originating from resident immune cells surrounding the postoperative lymphatic fluid, which are primarily involved in wound healing and inflammatory responses. We compared the 50 DEPs, including the top 25 upregulated and top 25 downregulated in melanoma lymphatic sEVs with those in the control group and examined alterations in the postoperative lymphatic fluid relative to the intraoperative control lymphatic fluid by conducting hierarchical clustering and heatmap analysis (Fig. 6*A*). The common set of upregulated proteins identified in both groups highlights key proteins associated with wound healing and inflammation, which are also known features of the PMN. Among all 50 DEPs, we found those that were commonly upregulated or downregulated in melanoma and postoperative lymphatic fluid as well as those uniquely modulated in the melanoma group only (Fig. 6*A*). Those commonly upregulated in the melanoma and postoperative samples emphasize the interferon gamma receptor–mediated PD-L1 checkpoint pathway (Supplemental Fig. S12). More significantly, the PD-L1 pathway is common, which is key in both wound healing and tumor promotion (39). This process connects interferon-gamma receptor 1 to Janus kinase 2 (JAK2), potentially resulting in the activation of PD-L1, a known immunosuppressive molecule that perpetuates both infection and the wound healing process (40).

### Melanoma-Specific sEV Cargo Proteins Are Involved in the Melanoma Progression

**Fig. 7:**
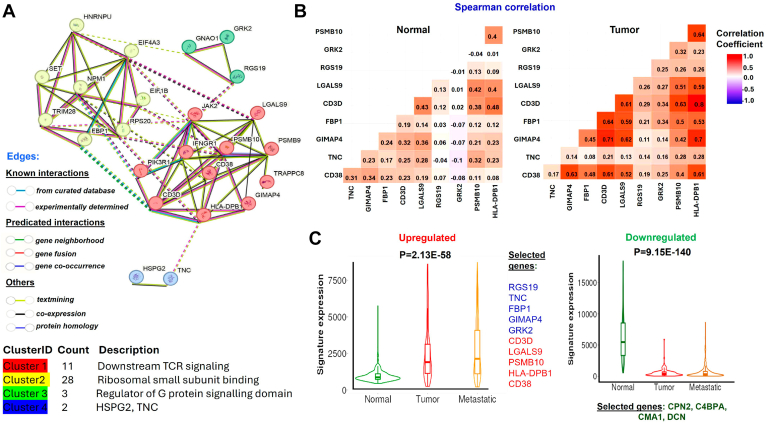
**Significance of identified proteins derived from melanoma afferent lymphatic sEVs in melanoma tumorigenesis.***A*, the proteomic network of all upregulated proteins found in melanoma lymphatic sEVs, when compared with controls, reveals four significant protein network clusters identified using the MCL clustering method. *B*, a Spearman's correlation matrix of the melanoma-associated upregulated proteins shows a higher correlation in melanoma tumor data compared with the normal dataset. *C*, gene signature analysis using RNA-Seq data in relation to the SKCM dataset of the highlighted upregulated and downregulated proteins in lymphatic sEVs of melanoma patients. MCL Markov Cluster algorithm; sEV, small extracellular vesicle; SKCM, skin cutaneous melanoma.

After analyzing all the upregulated and downregulated proteins in melanoma sEVs, we identified 11 proteins that may be directly associated with melanoma metastasis based on the comparison of data from TCGA. This emphasizes the role of sEVs from afferent lymphatic channel lymphatic fluid in initiating tumorigenic pathways in the recipient LN. The significance of these proteins was determined using TCGA-SKCM datasets using the TNM plotter web tool, which uses RNA-Seq data from normal tissues of noncancer patients (n = 474), tumor samples (n = 103), and metastatic cases (n = 368). Nine uniquely upregulated proteomic cargoes in melanoma, compared with postoperative lymphatic fluid or normal controls, signifies their significance in the gene expression at the metastatic progression (Fig. 6*B*). In addition, the reduced gene expression of two downregulated proteins was significantly associated with melanoma metastasis (Fig. 6*B*). Evaluating the DEPs of the upregulated proteins highlights the role of sEV cargo proteins in myeloid cell dysfunction and CD8 T-cell deactivation, among others supported by pathway analysis. In the proteomic network based on the Markov Cluster algorithm clustering method, the T-cell receptor is predominant (Fig. 7*A*). When we correlated the expression data at mRNA level, a stronger correlation of all 10 melanoma-specific cargoes was observed in the SKCM melanoma dataset compared with noncancer normal tissue (Fig. 7*B*). The TNM plotter–based signature panel analysis shows that upregulated proteins (RGS19, TNC, FBP1, GIMAP4, GRK2, CD3D, LGALS9, PSMB10, HLA-DPB1, and CD38) show high signature potential with a *p* value of 2.13 × 10^−58^. In addition, the downregulated candidates, including CPN2, C4BPA, CMA1, and DCN, also show high significance with a *p* value of 9.15 × 10^−140^. These findings highlight the relevance of these proteins as a signature panel in the context of melanoma progression (Fig. 7*C*).

### Expression Analysis of sEV Proteomic Cargoes in LN Sinuses and Blood Plasma sEVs in Melanoma Patients and Control Subjects Shows Clinical Significance

**Fig. 8:**
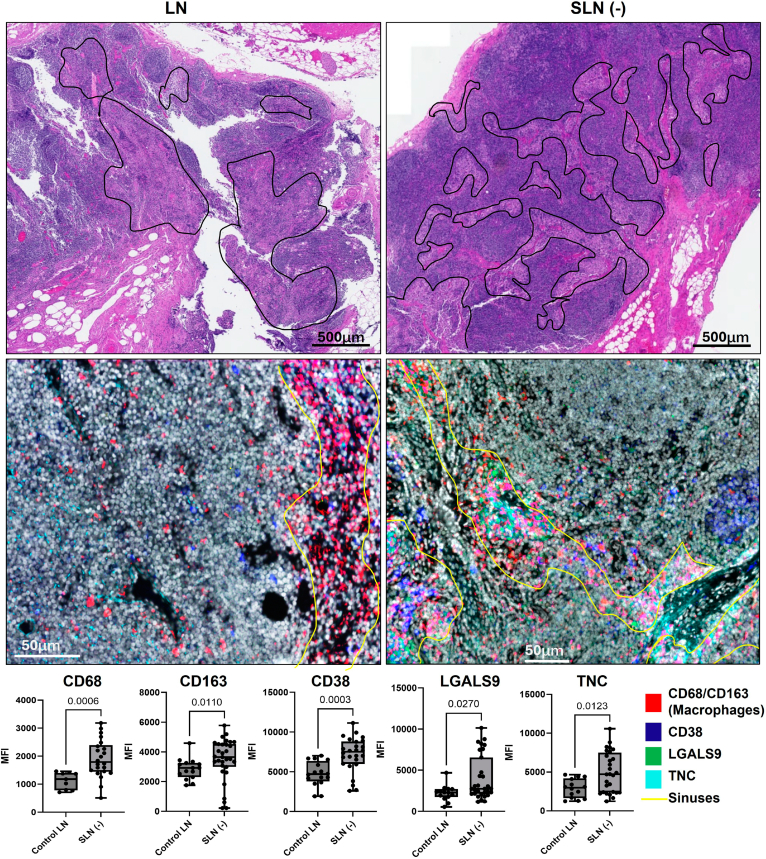
**Primary melanoma communicates sEV signals to facilitate a premetastatic niche within the sentinel lymph node (SLN).** Key melanoma-associated proteins, including CD38, LGALS9, and TNC, were analyzed in the sinuses of lymph nodes, comparing control and tumor-uninvolved SLN (−). The dot plot indicates the mean fluorescence intensity of these proteins, with each dot representing an individual field of view (FOV). sEV, small extracellular vesicle.

**Fig. 9:**
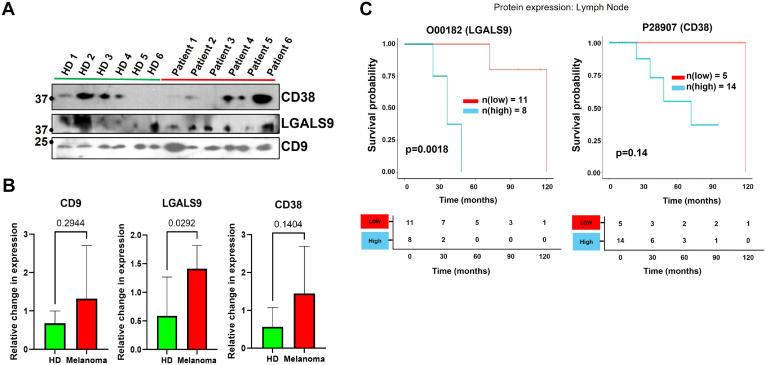
**Expression analysis and clinical significance of lymphatic sEV signals in melanoma tissues.***A*, the cargo of lymphatic sEVs, including CD38 and LGALS9, along with the universal sEVs marker CD9, was analyzed in the blood plasma of six melanoma patients and six healthy subjects. *B*, bar plots showing the relative changes in expression levels of CD9, LGALS9, and CD38 with total protein normalization. *C*, survival plot analysis based on the selected expression of proteins in lymph nodes affected by melanoma. sEV, small extracellular vesicle.

After comparative analysis of the expression profile of melanoma samples using various proteomic and transcriptomic datasets, as well as their relevance in postoperative samples. The proteins CD38, LGALS9, and TNC were selected for evaluation to determine whether lymphatic sinuses in melanoma patients exhibit elevated levels of these proteins compared with control LN sinuses. The early premetastatic modification of SLNs (“SLN–”) was found to be regulated by sEVs secreted by melanoma cells, rendering them suitable samples for studying the PMN. Sinuses were identified by a clinical pathologist (R.G.) based on chromogenic tissue sections derived from control LNs and uninvolved SLN (−), as these sinuses serve a critical function in the delivery of sEVs within the lymphatic system. In addition, these sinuses can be characterized by the macrophage’s dominant regions within the SLNs by histological observation. The annotated FOVs of the tissue samples had enriched sinuses were subjected to analysis, revealing an increase in macrophage markers (CD68 and CD163) within the LN sinuses of melanoma patients. Melanoma-derived EVs are known to expand the lymphatic sinus and generate premetastatic macrophages in the tumor-draining LN (41, 42). The MxIF analysis was conducted to compare the mean fluorescence intensity of each selected FOV in melanoma SLNs (−) with that of the control LN. The findings demonstrated that the expression levels of CD38, LGALS9, and TNC were significantly elevated in the sinus regions compared with the control LN sinus (Fig. 8). Furthermore, the upregulation of key melanoma-specific cargoes: LGALS9 (*p* = 0.029), CD3D (*p* = 0.097), and CD38 (*p* = 0.14) in the blood plasma sEVs of melanoma patients was noted in contrast to healthy donors (Fig. 9, *A* and *B* and Supplemental Fig. S13). Interestingly, the increased expression of LGALS9 in LN was also correlated with reduced overall survival in melanoma patients in the Cero dataset (*p* = 0.0018) (Fig. 9*C*). The overexpression of CD38, LGALS9, and TNC in lymphatic sEVs suggests their potential as biomarkers of early immune and stromal remodeling within the SLN. In which, CD38 indicates altered T- and B-cell activation (43), whereas LGALS9 (44) and TNC (45) reflect immunosuppressive signaling and ECM remodeling, respectively. Together, these sEV signatures may serve as a noninvasive indicator of PMN establishment. Clinical profiling of these proteins in sEVs could enable risk stratification by identifying melanoma patients with immunoregulatory or tumor-permissive SLNs, thereby guiding decisions on surveillance intensity or adjuvant therapy.

## Discussion

Tumor-derived sEVs influence the tumor microenvironment by facilitating intercellular communication, and they also have the potential to create a protumorigenic microenvironment. The cargoes within these sEVs are crucial for establishing the PMN, particularly in the LNs where initial melanoma cell metastasis occurs. Our research provided insight into sEVs in the lymphatic fluid, which are crucial in setting up metastasis in the SLN. We utilized quantitative proteomics to identify lymphatic sEV proteomes and to quantitatively assess the difference in protein levels between melanoma-associated and non–cancer-associated lymphatic sEVs. Our comparative study offers insight into deregulated proteomic cargoes into lymphatic sEVs in melanoma cases compared with controls, with samples collected from afferent lymphatic channels. It is important to note that lymphatic fluid from the afferent channel is responsible for bringing unprocessed antigens into the fluid from tissue, which differs from that in the efferent channel, which carries processed fluid away from the LNs. For this reason, afferent channel lymphatic fluid is crucial for our proteomic assessment of the melanoma-induced formation of PMN. The study encompasses in-depth features of lymphatic sEVs collected from inoperative LNs of melanoma and control cases as well as postoperative lymphatic fluid resulting from lymphadenectomy. The study provided compelling evidence of the dynamic nature of lymph through altering lymphatic sEVs, as postoperative lymphatic fluid sEV proteome composition significantly shifted from intraoperative lymphatic sEVs. Furthermore, postoperative lymphatic fluid sEVs may serve as a critical control for wound healing mechanisms implicated in lymphedema and lymphangiogenesis (46), key features of the PMN of SLN in melanoma (47). Hence, it helped us identify melanoma sEV cargoes linked to inflammatory responses and other oncogenic activities.

Lymphatic sEVs are crucial mediators in the transport of signaling molecules from peripheral tissues to SLNs, where they play a key role in intercellular communication and trigger systemic immunity in response to various stimuli carried by the lymphatic fluid. However, the procurement of these lymphatic sEVs is fraught with challenges, largely because of their elusive nature and the intricate dynamics of lymphatic circulation. As a result, there has been a significant gap in the exploration of omics technologies aimed at studying the composition and functionality of sEVs derived from the lymph of afferent lymphatic channels. An earlier study on the exudative seroma obtained from lymphadenectomy of patients with stage III melanoma shows enrichment in sEV proteins resembling melanoma progression (14). Lymphatic sEVs from afferent channels were found to be unique when compared with all reported proteomic cargoes of ExoCarta. Although lymphatic sEVs contain predominant established markers like tetraspanins, syntenin-1, and endosomal sorting complex required for transport proteins and match 94 of top 100 ExoCarta proteins, 595 unique proteins were identified (Fig. 3*A*). Furthermore, the Gene Ontology analysis of these unique proteins demonstrated significant enrichment for proteins associated with vesicles, immune system–processing activities, and chromatin assembly, and their protein family classification suggests their link to the regulatory function (Fig. 3*B*). Comparing with three previously reported lymphatic EV proteomic datasets (14, 15, 19), 1003 (19.6%) unique proteins were identified, along with 626 (12.2%) common proteins found in all datasets (Fig. 3*C* and Supplemental File S3). The IPA of DEPs links to increased cellular stress and injury pathways and a decrease in ECM organization (−log[*p* value] >7.0) (Supplemental Fig. S2) and associates with the activation of JAK–signal transducer and activator of transcription and other immune pathways are found to be associated with melanoma (Supplemental Figs. S3 and S4), which are involved during PMN formation as well as metastatic progression of cancer (48, 49). Immune-related pathways are among the keys to linking DEPs (Supplemental Fig. S5). The Kyoto Encyclopedia of Genes and Genomes pathway analysis of upregulated proteins in the melanoma lymphatic sEVs highlights the PD1 and PDL1 checkpoint pathway and Th1/Th2 cell differentiation pathway (Fig. 4*C*). However, the top hits among the downregulated proteins are associated with protein digestion and absorption as well as ECM–receptor interaction proteins (Fig. 4*C*). In recent years, PD1/PDL1 has been considered as an important target for immunotherapies against melanoma, as these proteins play a significant role in resisting T-cell immune function (50, 51). Studies have also shown that Th1/Th2 imbalance is also a major issue in melanoma progression (52, 53). In our previous studies, we found that melanoma-derived EVs increase the release of immunosuppressive cytokines by macrophages, potentially fostering the development of a PMN in the SLN (54). It has also been observed that the cargo of melanoma cell EVs includes cellular clones that possess distinctly different metastatic potentials, which can facilitate cell migration by transferring EV contents that operate within the same physiological signaling networks (55). Our present study shows that these crucial signals are directly regulated by melanoma sEVs in the lymphatic system, which contributes to the suppression of SLN immunity. In addition, the structural remodeling of SLN, particularly the expansion of the LN, is a key mechanism that prepares SLN for metastasis (56). Previous studies have provided evidence that lymphatic drainage is involved in this process (57). Many of these downregulated proteomic cargoes in melanoma sEVs, including collagens (COL1A1, COL3A1, COL6A1–A3, and COL14A1), matrix-associated proteoglycans (DCN, LUM, FMOD, OGN, and PRELP), and structural regulators (TNXB and PCOLCE), are key components of ECM organization and tensile strength (58). Moreover, SOD3 (59), SELENOP (60), and PROS1 (61) can also regulate ECM integrity. It is more likely that depleted levels of these cargoes in lymphatic sEVs in melanoma could weaken ECM integrity, which can facilitate cellular or sEV transport (62, 63). Collectively, these findings reveal that biophysical modifications in the LN may arise not only from the gain of proremodeling factors of SLN but also from the loss of structural and homeostatic proteins in the lymphatic sEVs that normally preserve tissue stability.

Among the downregulated proteins, paraoxonase 1 (PON1), which protects against oxidative stress (64), and C4BPA, which inhibits cancer progression by promoting antitumor T cells (65), are notable. The decreased proteomic cargoes in melanoma highlight complement pathways, which are known to play a role in innate immunity and influence cancer progression (34).

Cancer and wound healing share common hallmarks, with compromised regulation of wound healing pathways facilitating cancer progression (66). This interplay involves the commandeering of wound healing "master regulators," affecting treatment strategies for chronic wounds and cancer alike (67). The inflammatory response during wound healing can accelerate the growth of preneoplastic cells and promote tumor colonization (68, 69). In addition, a postoperative lymphatic fluid, which is a pocket of lymphatic fluid from disrupted drainage, can serve as a model in wound healing studies (70). Our studies have identified key pathways that mediate tumor-promoting functions, such as the INFR1–JAK2 and PDL1 pathways (71). Impaired interferon signaling is a common immune defect in many human cancers (72). PD-L1 is well reported to be induced by interferon gamma signaling in melanoma cells (73). These pathways serve as crucial signals in the wound healing process as well (74). The upregulated proteins show strong inflammatory signals that melanoma secretes to develop a PMN in SLN (Fig. 6 and Supplmemental Fig. S12). We identified proteins that are uniquely overexpressed in melanoma lymphatics, which did not change in postoperative lymphatic fluid compared with the control. From this analysis, 11 proteins were significantly associated with their roles in melanoma metastasis when compared with SKCM RNA-Seq data (Fig. 6*B*). The SKCM data also show evidence of strong correlation of 10 candidates (CD3D, FBP1, GIMAP4, GRK2, LGALS9, PSMB10, RGS19, TNC, HLA-DPB1, and CD38) in melanoma compared with noncancer normal tissue. Interestingly, most of these proteins are well known in multiple cancers, including melanoma.

Melanoma-derived sEVs are known to modify SLNs prior to colonization, rendering them critical to the study of early metastatic events. To determine the abundance of the selected proteomic signatures in the sinuses of the LN, we employed pathologist-guided MxIF analysis to compare fluorescence intensity between negative melanoma SLNs and control LN, and we detected significantly higher levels of CD38 (*p* = 0.0003), LGALS9 (*p* = 0.027), and TNC (*p* = 0.012) in the sinuses of melanoma patients (Fig. 8). We detected upregulated LGALS9 (*p* = 0.029) and CD38 (*p* = 0.14) in the sEV cargoes in the blood plasma sEVs of melanoma patients than in healthy donors (Fig. 9, *A* and *B*). We also found a higher level of additional sEV cargo CD3D in the blood plasma of melanoma (*p* = 0.097) (Supplemental Fig. S13). Interestingly, higher levels of LGALS9 in the LN tissues also correlated with lower overall survival in melanoma patients, based on the Cero dataset data (*p* = 0.0018) (Fig. 9*C*). These results demonstrate the potential biomarker capability of these lymphatic sEV proteomic cargos during PMN formation. Moreover, LGALS9 expression correlates with poor prognosis in multiple human cancers (75). Our earlier study shows that LGALS9 binds with myeloid cells and promotes a tumor-supportive microenvironment (76). CD38 is a protumoral factor involved in the stromal regulation for angiogenesis and metastasis (77) and is commonly reported in lung to brain metastasis (78). CD3D is well reported in gastric cancer and melanoma for its role in immune cell regulation (79, 80). Moreover, TNC is reported as a critical regulator of melanoma progression (81, 82). The overexpression of CD3D and CD38 in sEV of melanoma patient lymphatics reflects critical changes in immune cell composition and activity within the SLN. CD3D, a marker of T cells relevant to adaptive immunity as well as associated with a favorable prognosis of melanoma (83), whereas CD38, which regulates NAD^+^ metabolism, marks activated B cells and plasma cells (84). This clearly shows an altered representation in lymphatic sEVs likely mirrors shifts in immune cell composition or activation within the premetastatic SLN, rather than changes intrinsic to EV biogenesis. These sEV cargoes are found in higher abundance in the melanoma sinus macrophages, which may be instrumental in the early immune remodeling of PMN development. It is more likely that sEVs interfere with macrophage functions and disrupt T–B-cell interactions (85). Previous studies indicate that macrophages play a key role in processing EVs and influencing local immune responses (54, 86). Together, these changes help drive early transformation of the SLN and the formation of a PMN. Detecting these sEV-associated proteomic cargoes could provide a noninvasive window into SLN immune remodeling, offering the potential to guide patient risk assessment, surveillance, and therapeutic decisions (87).

In summary, this study presents a comparative analysis of sEV proteomic alterations in the afferent channel, focusing on melanoma patients and control subjects, as well as postoperative lymphatic fluid. By analyzing sEVs collected within the afferent lymphatics, we conducted a detailed proteomic comparison with other proteomic and TCGA-SKCM datasets, revealing the significance of proteomic cargoes in melanoma progression. Our findings show that melanoma-associated sEVs contain key proteins that may regulate SLN immunity through various signaling pathways, affecting both innate and adaptive immune responses. Furthermore, these sEVs carry unique immune-related as well as ECM-organizing proteins that play a critical role in establishing a PMN, suggesting that they modify SLN to facilitate metastasis. We emphasize comparing our data with previously published datasets in studying protein expressions and their correlation with clinical outcomes in melanoma. Our analysis has determined that sEVs within melanoma lymphatics may contribute to the establishment of a protumorigenic environment in SLN. Among key sEV cargo proteins, LGALS9, CD38, and TNC have been identified as upregulated in the lymphatic sinus during the development of a PMN. The proteomic signatures of these sEV cargoes could serve as clinically relevant biomarkers for the PMN for melanoma; further validation with a larger dataset of clinical samples could be vital before confirming their clinical utility.

## Data Availability

The MS proteomics data have been deposited to the ProteomeXchange Consortium *via* the PRIDE (23) partner repository with the dataset identifier PXD063898 and 10.6019/PXD063898.

## Supplemental Data

This article contains supplemental data (14, 24, 26).

## Conflict of Interest

The authors declare no competing interests.
