# Supplementary material for: Proteomic Analysis of Small Extracellular Vesicles From Lymphatic Affluents in Developing Premetastatic Niche in Melanoma
Source: Mol Cell Proteomics. 2025 Nov 19;25(1):101472. doi: 10.1016/j.mcpro.2025.101472 (PMC12794256; doi:10.1016/j.mcpro.2025.101472)
Supplement: Supplemental Figures [file mmc1.pptx]

## Slide 1
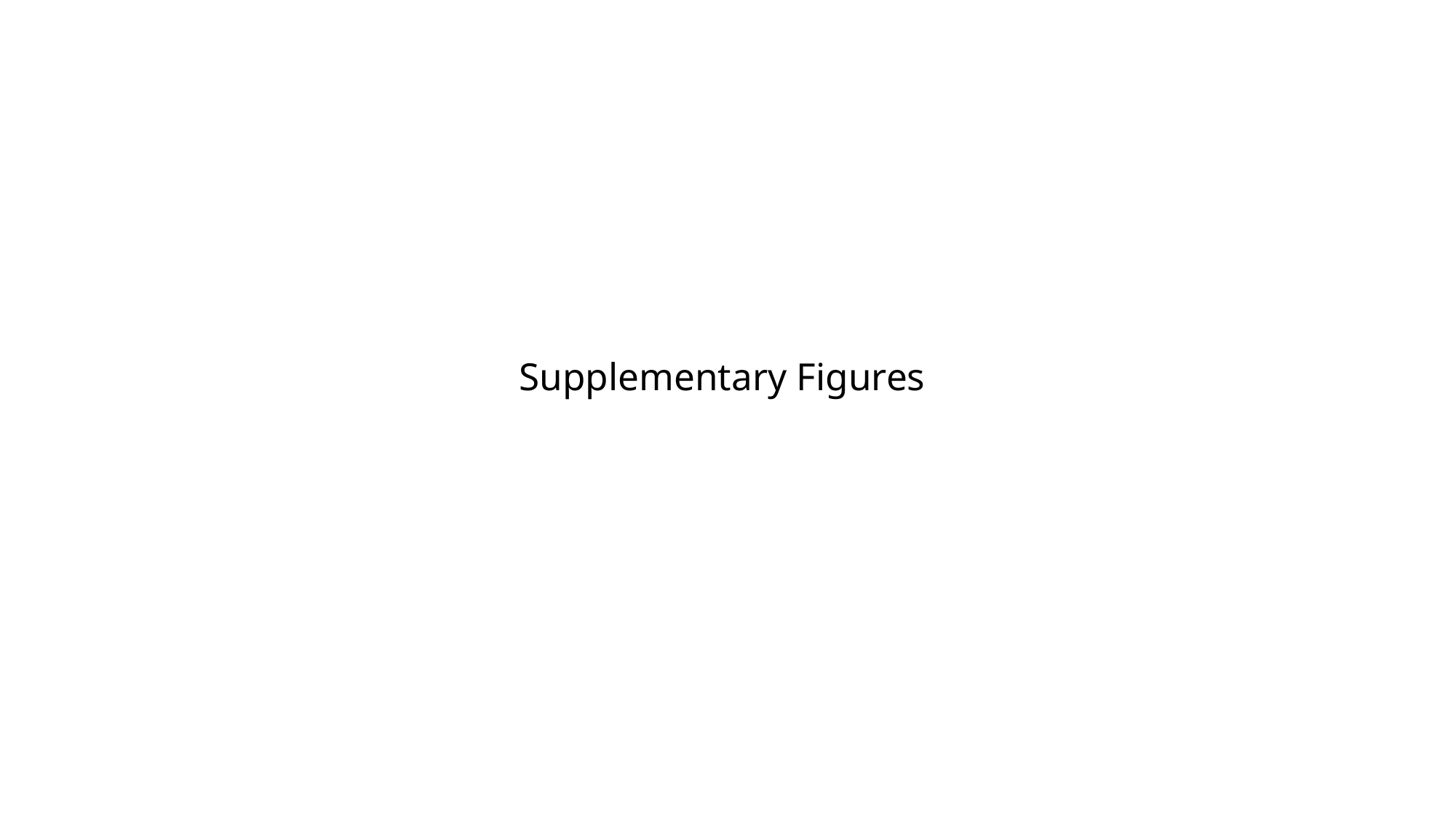

Supplementary Figures

## Slide 2
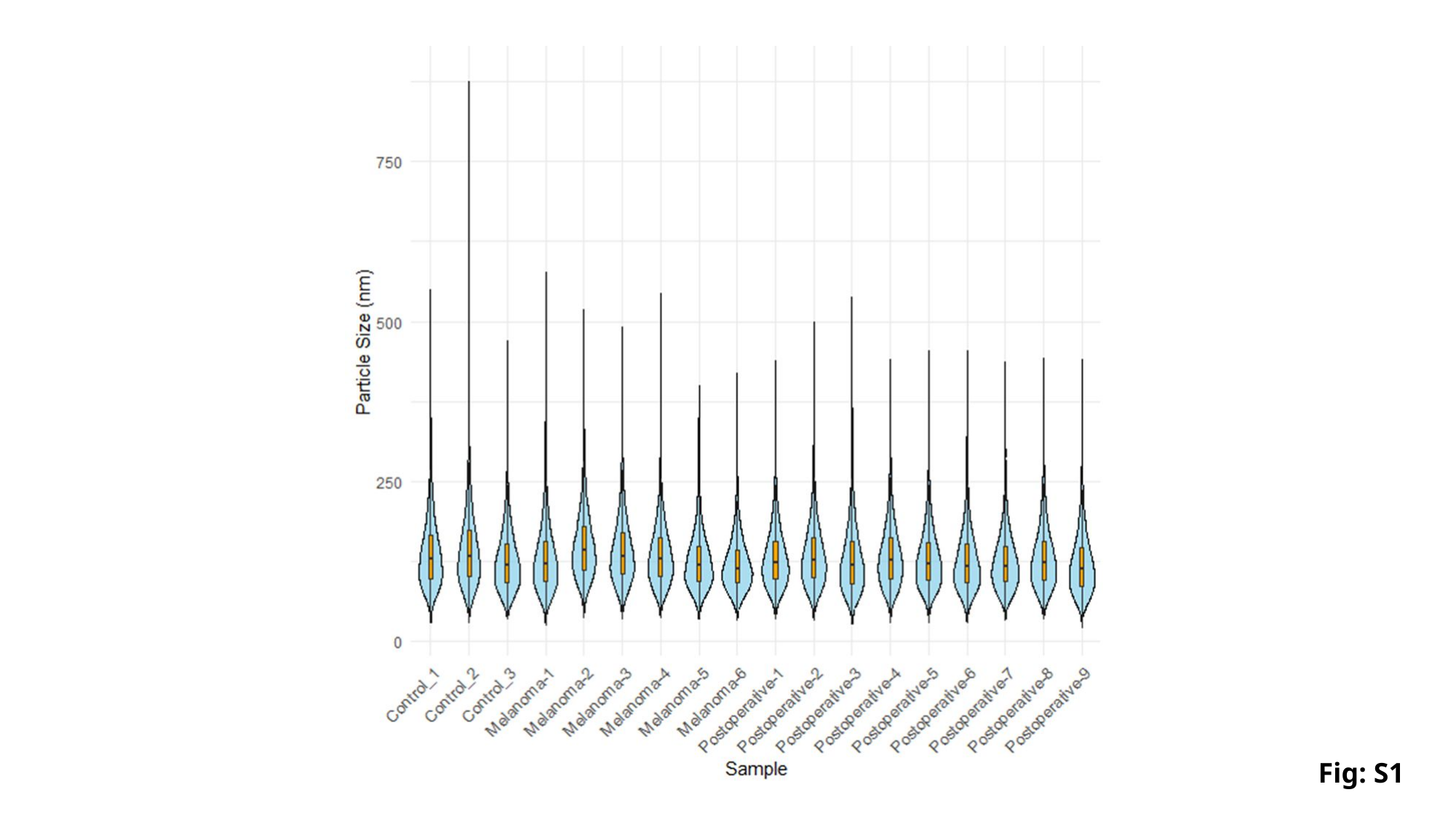

Fig: S1

## Slide 3
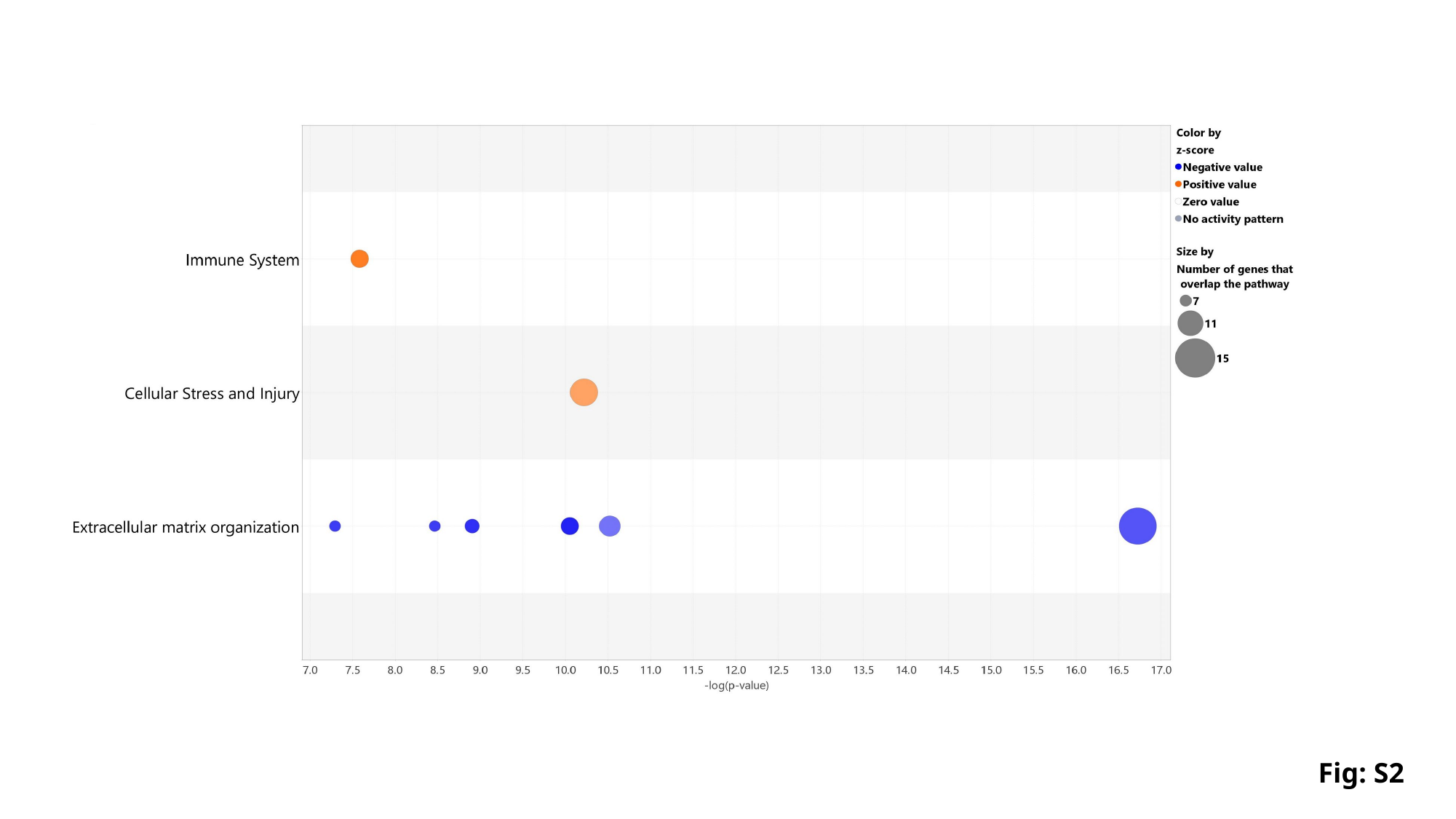

Fig: S2

## Slide 4
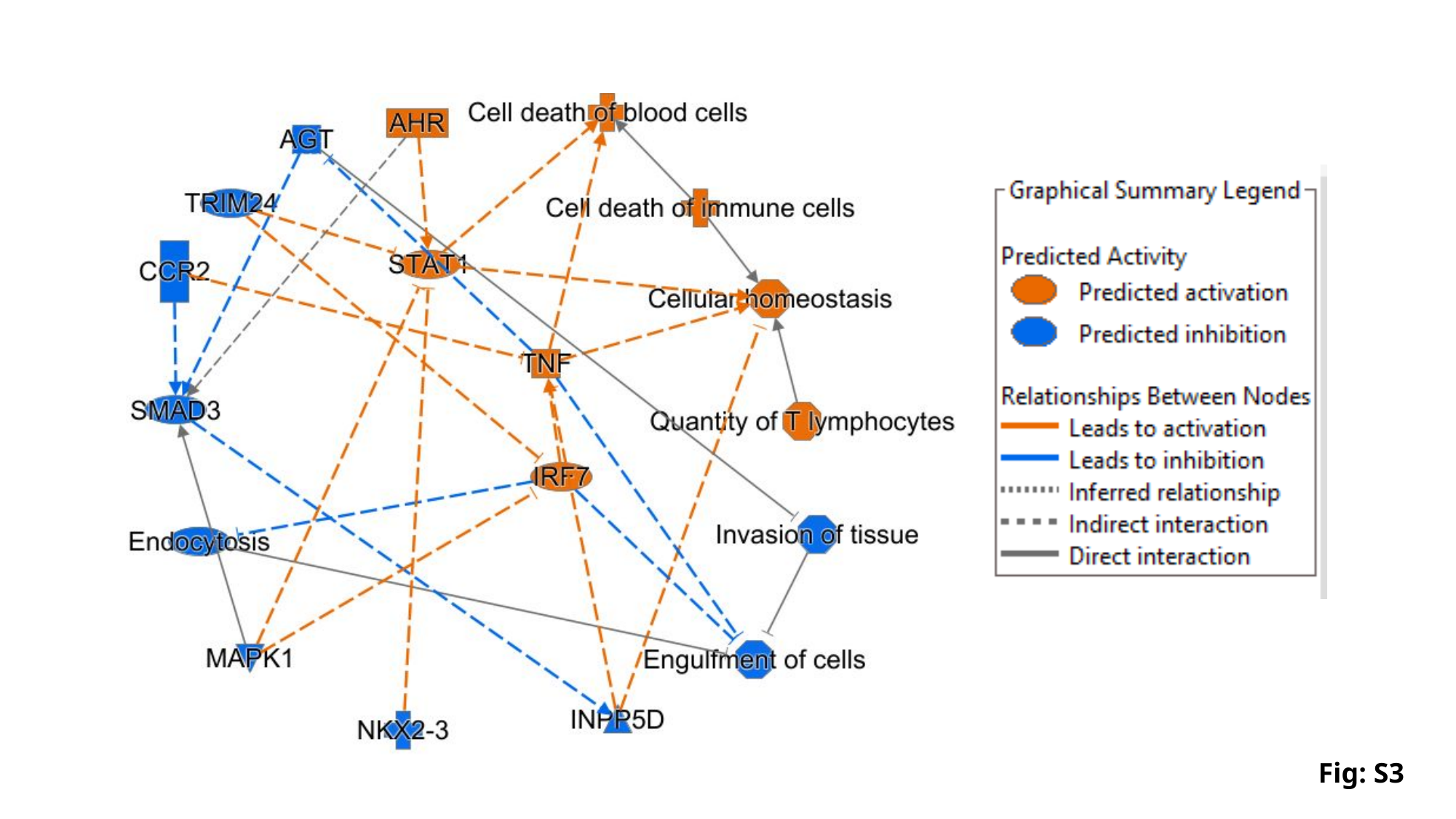

Fig: S3

## Slide 5
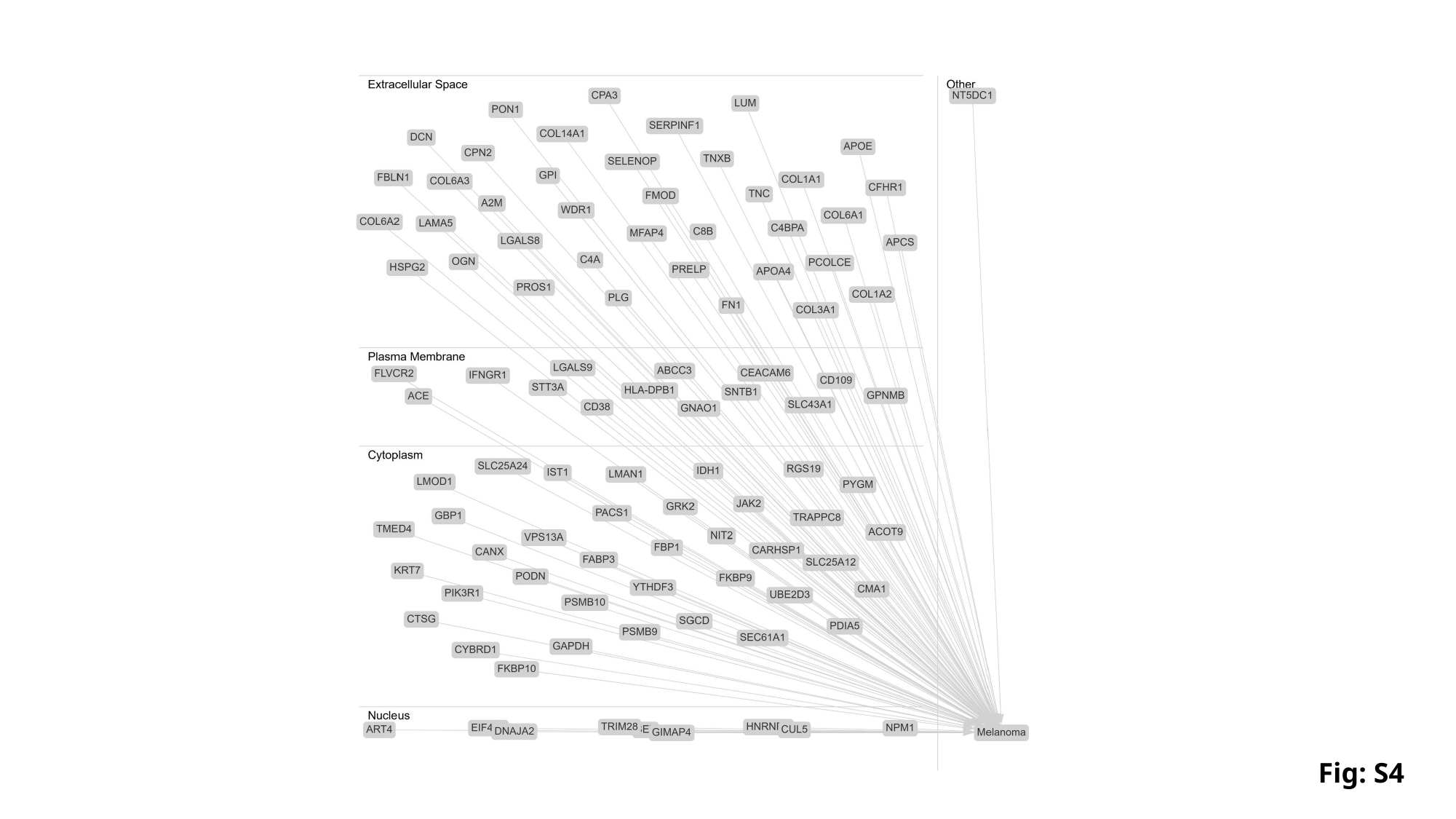

Fig: S4

## Slide 6
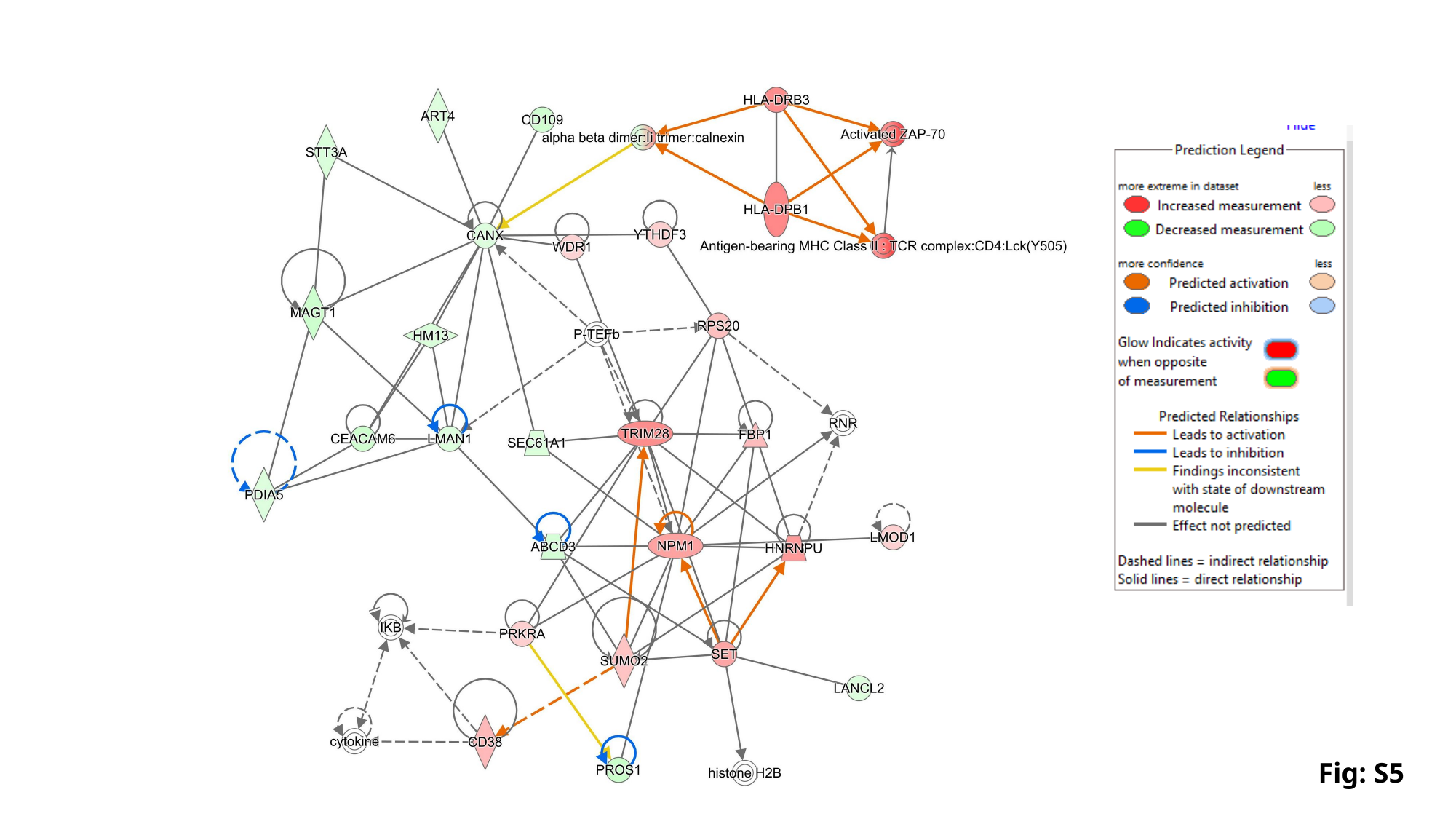

Fig: S5

## Slide 7
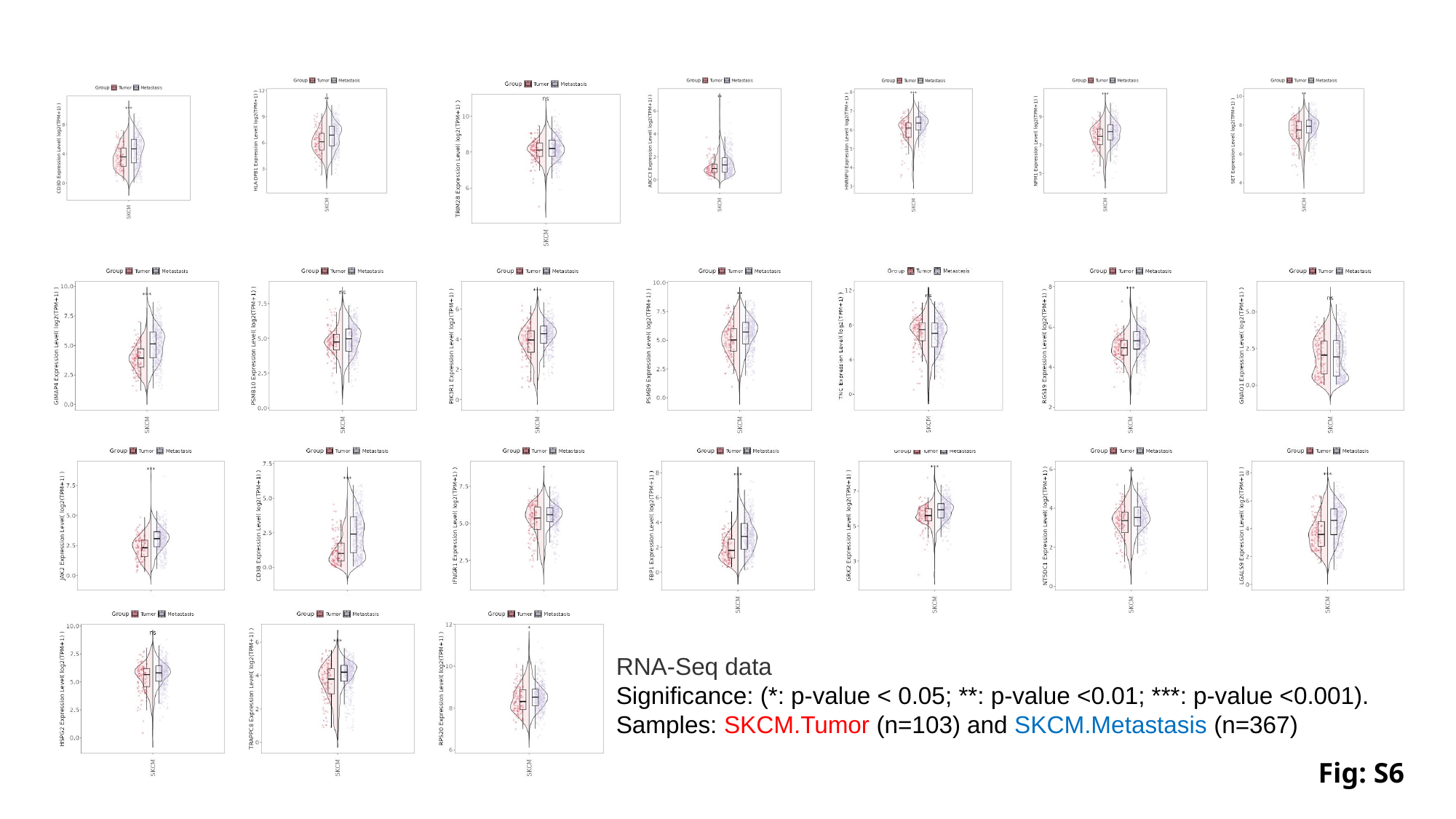

RNA-Seq data
Significance: (*: p-value < 0.05; **: p-value <0.01; ***: p-value <0.001).
Samples: SKCM.Tumor (n=103) and SKCM.Metastasis (n=367)
Fig: S6

## Slide 8
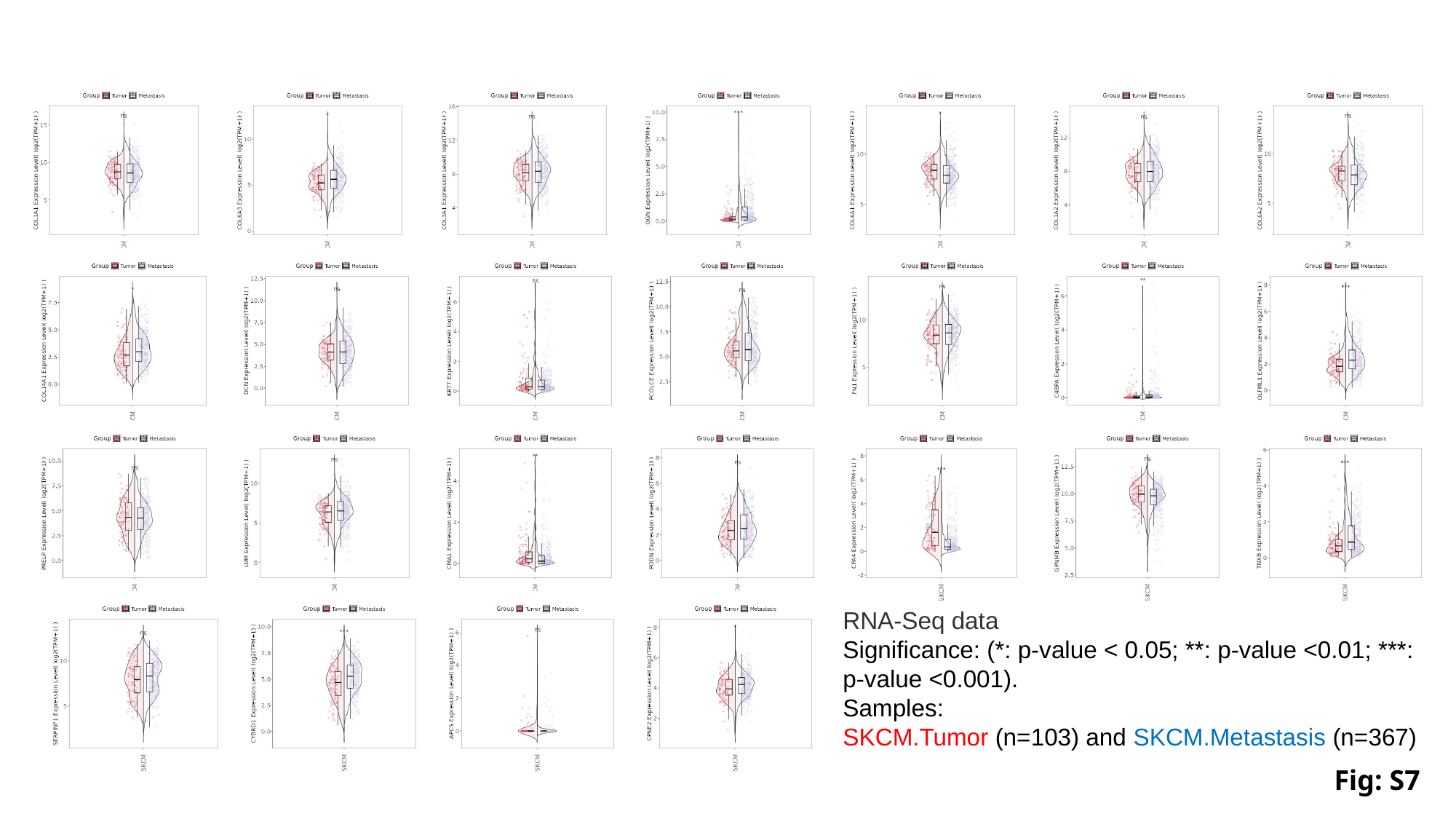

RNA-Seq data
Significance: (*: p-value < 0.05; **: p-value <0.01; ***: p-value <0.001).
Samples:
SKCM.Tumor (n=103) and SKCM.Metastasis (n=367)
Fig: S7

## Slide 9
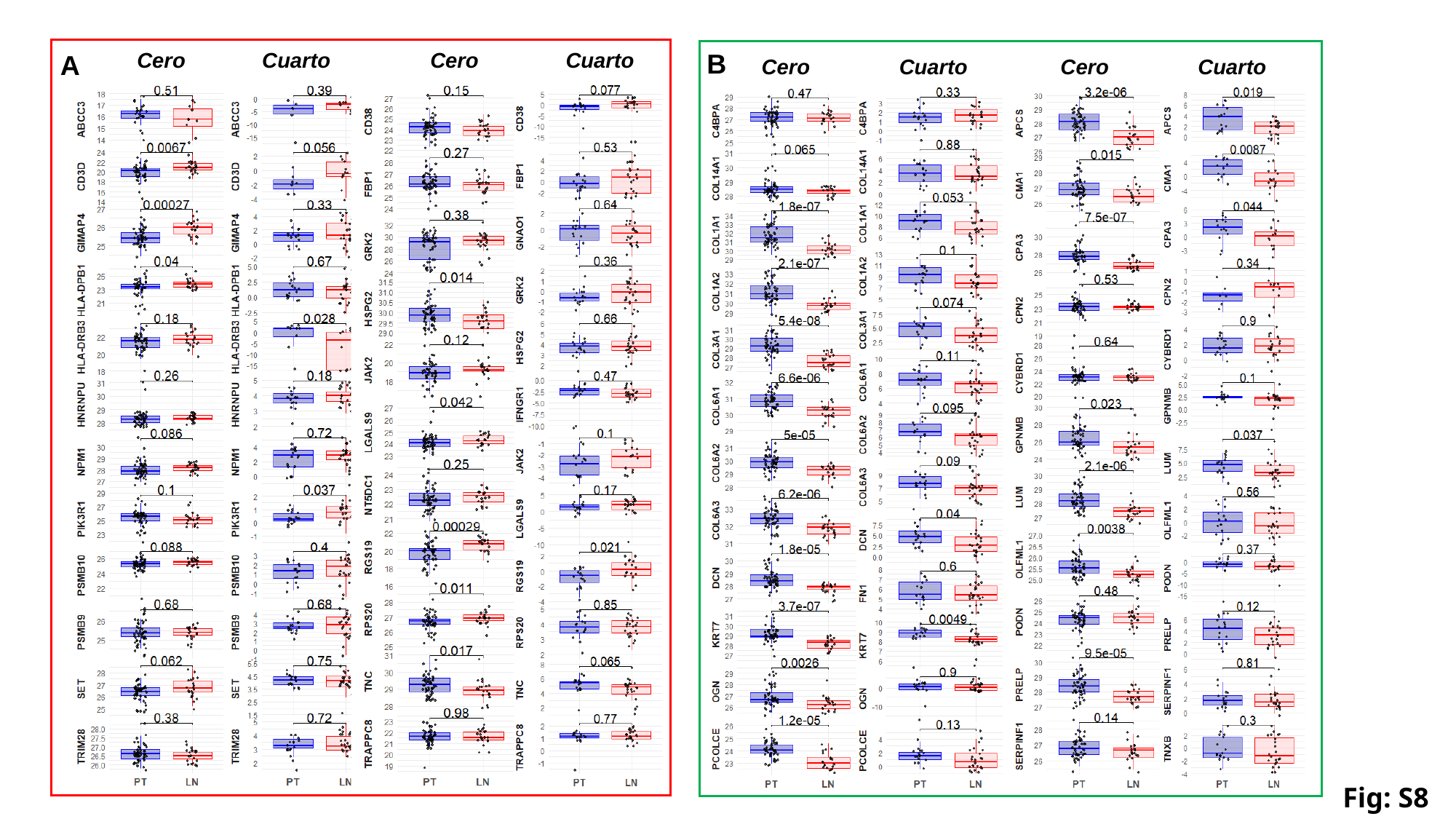

Cero
Cuarto
Cuarto
Cero
B
Cero
Cuarto
Cero
Cuarto
A
Fig: S8

## Slide 10
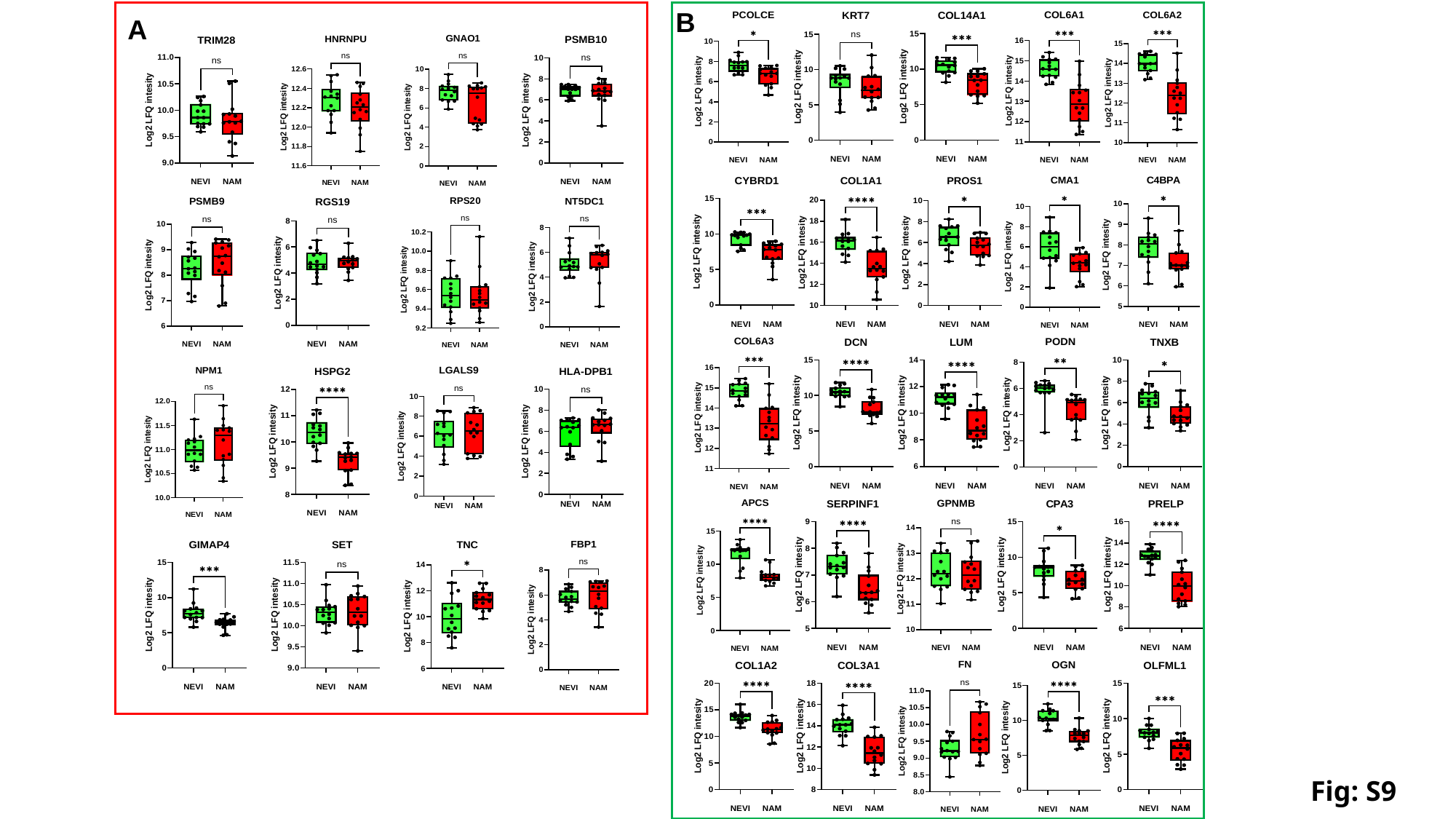

B
A
Fig: S9

## Slide 11
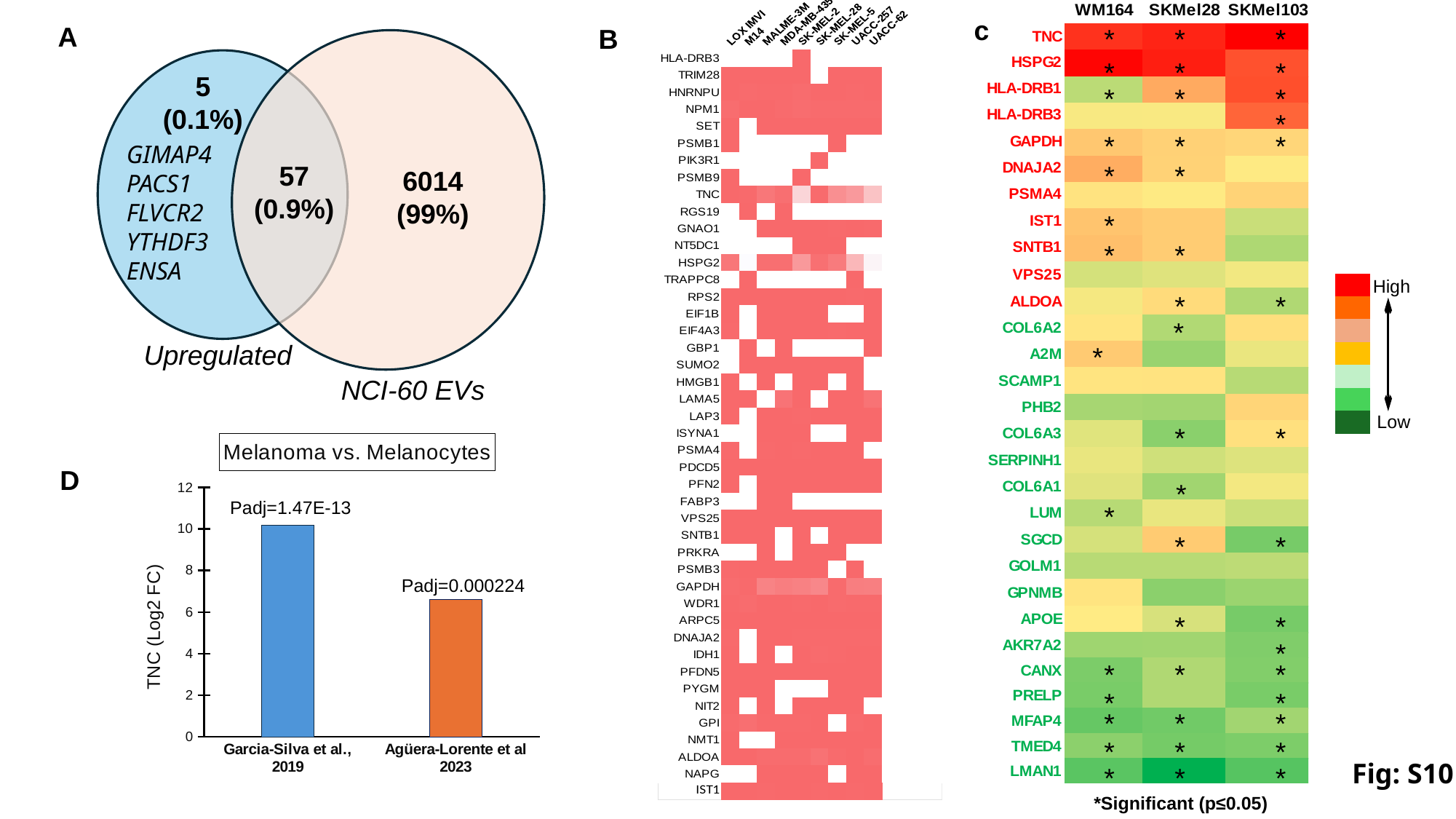

*
*
*
*
*
*
*
*
*
*
*
*
*
*
*
*
*
*
*
*
*
*
*
*
*
*
*
*
*
*
*
*
*
*
*
*
*
*
*
*
*
*
*
*
*
*Significant (p≤0.05)
c
A
B
5
(0.1%)
57
(0.9%)
6014
(99%)
Upregulated
NCI-60 EVs
GIMAP4
PACS1
FLVCR2
YTHDF3
ENSA
High
Low
### Chart: Melanoma vs. Melanocytes
| Category | Melanocyte_vs_Melanoma |
|---|---|
| Garcia-Silva et al., 2019 | 10.2 |
| Agüera-Lorente et al 2023 | 6.61 |Padj=1.47E-13
Padj=0.000224
D
Fig: S10

## Slide 12
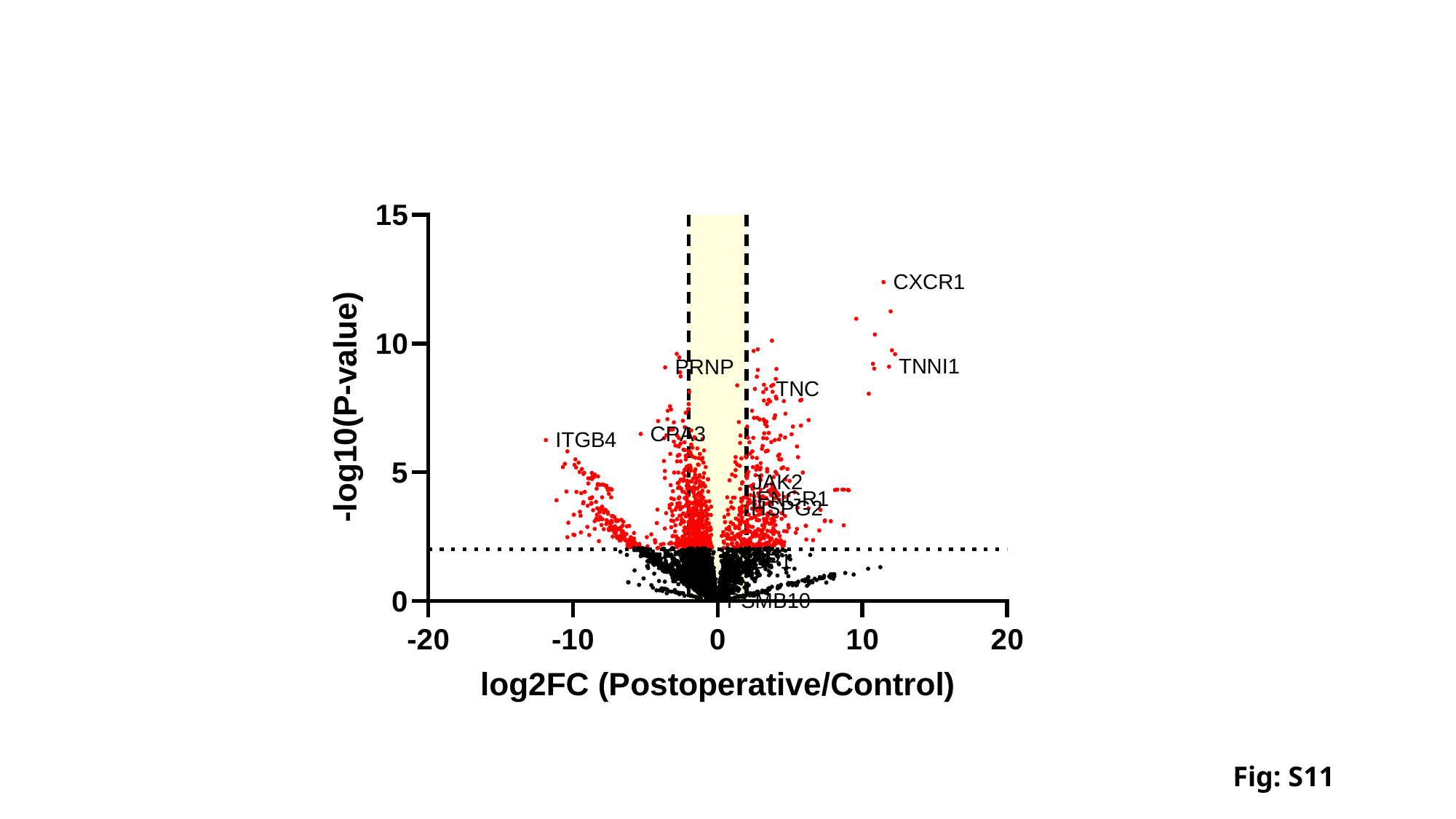

Fig: S11

## Slide 13
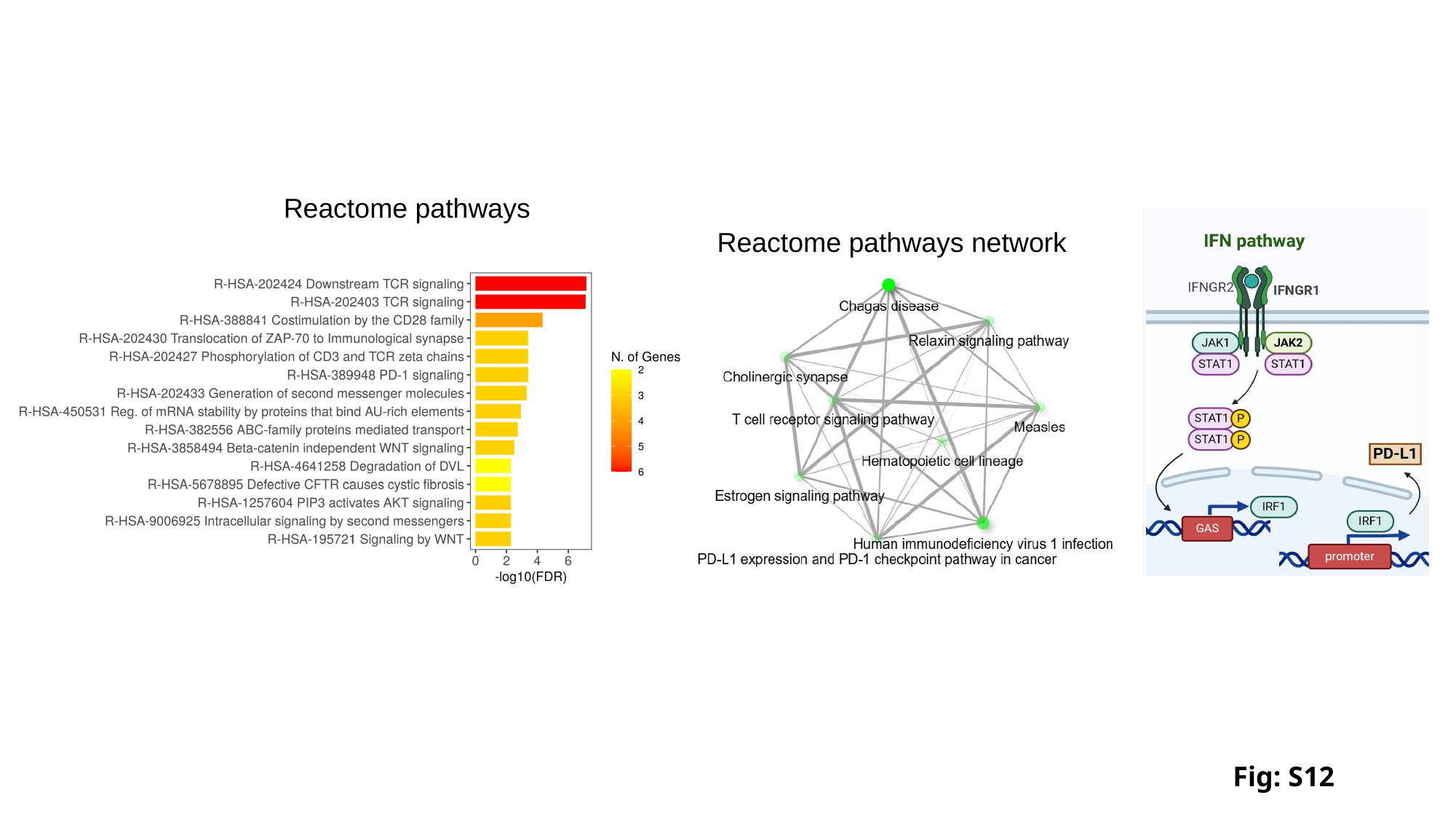

Reactome pathways
Reactome pathways network
Fig: S12

## Slide 14
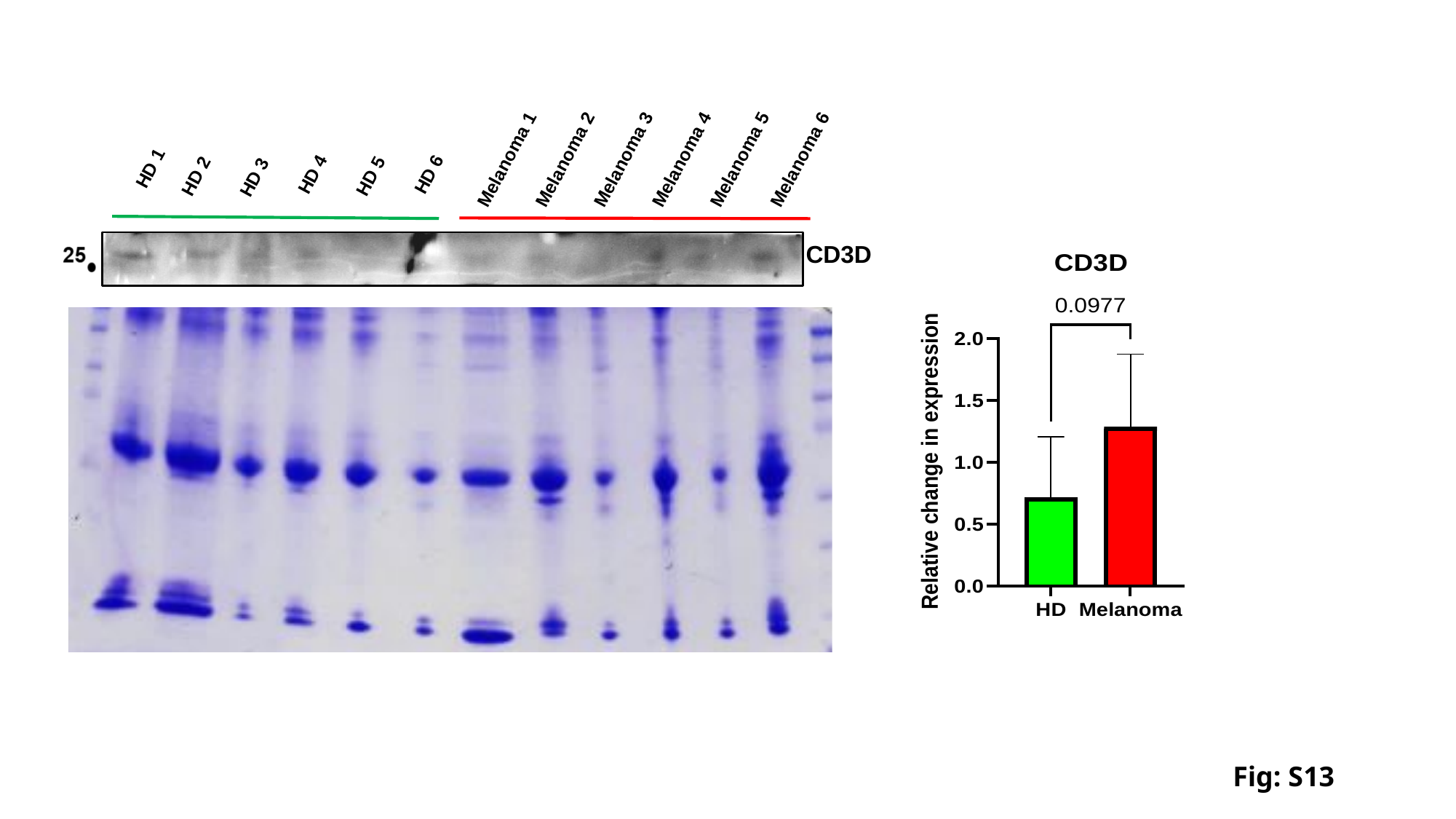

Melanoma 1
Melanoma 2
Melanoma 3
Melanoma 4
Melanoma 5
Melanoma 6
HD 1
HD 6
HD 4
HD 5
HD 2
HD 3
CD3D
Fig: S13
