# Supplementary material for: Proteomic Analysis of Small Extracellular Vesicles From Lymphatic Affluents in Developing Premetastatic Niche in Melanoma
Source: Mol Cell Proteomics. 2025 Nov 19;25(1):101472. doi: 10.1016/j.mcpro.2025.101472 (PMC12794256; doi:10.1016/j.mcpro.2025.101472)

**Proteomic analysis of small extracellular vesicles from lymphatic affluents in developing premetastatic niche in melanoma.**

Shankar Suman^1^, Liyi Geng^1^, Wendy K. Nevala^1^, Raymond Moore^2^, Chathu Atherton^1^, Xiaowei Zhao^3^, Jaeyun Sung^2^, Ray Guo^4^, James W. Jakub^5^, Richard K. Kandasamy^6,7,8^, Sarah A. McLaughlin^5^, Akhilesh Pandey^6,8^, and Svetomir N. Markovic^1,7^

**Supplementary data**

**Files:**

**Supplementary File 1:** List of identified proteomic cargoes in the lymphatic sEVs

**Supplementary File 2:** Unique proteins in lymphatic sEVs in our dataset compared to ExoCarta.

**Supplementary File 3:** List of unique identified proteomic cargoes in our lymphatic sEVs compared to other published datasets.

**Supplementary File 4:** Significantly modulated proteins in melanoma sEVs

**Supplementary Figures:**

**S1:** Violin plot of sEV particle size of all samples used in the proteomics experiment.

**S2:** Bubble chart showing canonical pathway categories of upregulated ann downregulated proteins in melanoma derived lymphatic small extracellular vesicles (sEVs)

**S3:** Pathway network of all modulated proteins in melanoma derived lymphatic small extracellular vesicles (sEVs)

**S4:** Modulated sEVs cargo proteins and their cellular localization of melanoma associated proteins.

**S5:** IPA pathway network of modulated proteins

**S6:** Gene expression analysis in tumor and metastatic melanoma samples of top 25 upregulated proteins in melanoma-derived sEVs in TCGA-SKCM dataset using the TIMER 3 platform.

**S7**: Gene expression analysis in tumor and metastatic melanoma samples of top 25 downregulated proteins in melanoma-derived sEVs in TCGA-SKCM dataset using the TIMER 3 platform.

**S8:** Expression of top 25 modulated proteins in primary melanoma (PT) and lymph node metastatic tumor (LN) and in Cero and Curato datasets A. Upregulated B. Top 25 downregulated

**S9:** Comparison of modulated proteins in Nevi and nevus-associated melanoma (NAM) in Naimy et al dataset. A. matched upregulated proteins B. matched downregulated proteins.

**S10**: A. Venn diagram illustrating 57 out of 62 upregulated proteins present in NCI-60 extracellular vehicles (EVs). B. Presence of identified cargo proteins in various melanoma cell lines. C. A comparison of upregulated proteins (highlighted in red) and downregulated proteins (highlighted in green) from our dataset, showing their expression levels in melanoma cell-derived EVs compared to those in melanocyte EVs. D. Log2 FC in melanoma vs melanocytes in two studies.

**S11**: Volcano plot of exosomal proteomic cargo altered in postoperative lymph compared to intraoperative control afferent channel lymph.

**S12**. Reactome pathways enrichment analysis of all upregulated and downregulated proteins in postoperative and melanoma lymphatic exosomes. Network analysis emphasizes the interferon gamma receptor (IFNGR) mediated PDL1 checkpoint pathway within the results.

**S13.** CD3D expression analysis in the plasma sEVs from melanoma patients (n=6) and healthy donors (n=6). Bar chart showing the relative changes in expression levels with total protein normalization with SDS-gel.

**Supplementary Table1:** Clinicopathological details of lymphatic fluid samples

|  | **Control** | **Melanoma** | **Postoperative** |
| --- | --- | --- | --- |
| No of subjects | 3 | 6 | 9 |
| Age | 58 ± 14.7 | 44 ± 18.9 | 58.5 ± 15.7 |
| Race | White:3 | White:6 | White: 8  African American: 1 |
| Surgery:  ALND  (Axillary lymph node dissection) | Mastectomy + ALND: 3 | ALND:6 | Mastectomy + ALND: 7  ALND only: 2 |
| SLN type | NA | SLN (-): 4  SLN (+): 2 | NA |
| T Stage at Sx | NA | pT2a:3  pT3b:1  pT4a:1 | NA |
| N Stage at Sx | NA | N0:4  N1a: 2 | NA |
| Breslow thickness (mm) | NA | 3.08 ± 3.4 | NA |

**Supplementary Table2**: Clinicopathological details of lymph node tissues for MxIF analysis

| **Cases** | **#1** | **#2** | **#3** | **#4** |
| --- | --- | --- | --- | --- |
| Primary  diagnosis | Melanoma | Melanoma | Benign salivary gland tissue | Benign gall bladder |
| Tissue types-LN | SLN negative | SLN negative | Control LN | Control LN |
| Age | 81 | 48 | 61 | 43 |
| Sex | M | F | M | M |
| Location | Left axillary | Left axillary | Left superficial parotid gland | Gallbladder cystic duct |
| Breslow thickness | 10mm | 1.39mm | NA | NA |
| Pathological Tumor stage | T4b | T2a | NA | NA |

**Supplementary Table3:** Clinicopathological details of blood plasma samples

|  | **Healthy Donors** | **Melanoma** |
| --- | --- | --- |
| No of subjects | 6 | 6 |
| Age range | 23-45 years | 51-82 years |
| Sex | Male: 3  Female: 3 | Male:3  Female: 3 |

**Supplementary Table 4:** List of antibodies used in MxIF analysis

| **Antibody:** | **Company:** | **Catalog Number:** |
| --- | --- | --- |
| Anti-CD68 antibody [KP1] | abcam | ab233172 |
| Anti-CD163 Antibody [EDHu-1] | BioRad | MCA1853 |
| Anti-Galectin-9 antibody [9S2-1] | Biolegend | 650702 |
| Anti-Tenascin C antibody [EPR4219] | abcam | ab215369 |
| Anti-CD38 antibody [5C5C3] | abcam | ab204940 |

**Supplementary Table 5**: Cellular component and PTM analysis of modulated proteins in melanoma lymphatic exosomes

|  | **Count** | **FDR** | **Genes** |
| --- | --- | --- | --- |
| **Upregulated proteins: PTMs** | | | |
| KW-0597~Phosphoprotein | 20 | 0.210860454 | ABCC3, NPM1, RGS19, SET, IFNGR1, EIF4A3, TNC, HNRNPU, GIMAP4, TRAPPC8, PIK3R1, CD3D, PSMB10, GRK2, TRIM28, HLA-DPB1, RPS20, JAK2, EIF1B, FBP1 |
| KW-0007~Acetylation | 12 | 0.172255116 | NT5DC1, NPM1, SET, TRIM28, EIF4A3, HNRNPU, RPS20, PIK3R1, EIF1B, FBP1, PSMB10, PSMB9 |
| KW-0832~Ubl conjugation | 10 | 0.172255116 | NPM1, SET, TRIM28, IFNGR1, EIF4A3, HNRNPU, RPS20, PIK3R1, JAK2, HLA-DRB3 |
| KW-0013~ADP-ribosylation | 4 | 0.029787113 | GNAO1, NPM1, TRIM28, HNRNPU |
| **Upregulated proteins: Cellular components** | | | |
| GO:0016020~membrane | 18 | 0.004167623 | ABCC3, NPM1, RGS19, IFNGR1, EIF4A3, TNC, HNRNPU, PIK3R1, HSPG2, CD3D, GNAO1, NT5DC1, GRK2, HLA-DPB1, CD38, RPS20, JAK2, HLA-DRB3 |
| GO:0005886~plasma membrane | 12 | 0.446550097 | GNAO1, ABCC3, GRK2, RGS19, IFNGR1, HLA-DPB1, CD38, PIK3R1, JAK2, HLA-DRB3, CD3D, HSPG2 |
| KW-0472~Membrane | 12 | 1 | GNAO1, ABCC3, NT5DC1, GRK2, RGS19, IFNGR1, HLA-DPB1, HNRNPU, CD38, JAK2, HLA-DRB3, CD3D |
| CARBOHYD: N-linked (GlcNAc...) asparagine | 8 | 1 | ABCC3, IFNGR1, TNC, HLA-DPB1, CD38, HLA-DRB3, CD3D, HSPG2 |
| **Downregulated proteins: PTMs** | | | |
| KW-0325~Glycoprotein | 41 | 1.54E-09 | APCS, FKBP10, TNXB, COL14A1, PROS1, PON1, SLC43A1, C4BPA, PLG, PRELP, MAGT1, C8B, CPN2, C4A, GPNMB, OLFML1, CTSG, TMED4, LUM, CMA1, SERPINF1, FN1, KRT7, PCOLCE, PODN, SOD3, DCN, COL1A1, MFAP4, COL3A1, COL1A2, CFHR1, CEACAM6, COL6A2, CD109, COL6A1, SELENOP, OGN, COL6A3, FKBP9, FMOD |
| KW-1015~Disulfide bond | 31 | 2.20E-05 | APCS, TNXB, COL14A1, PROS1, PON1, C4BPA, PLG, PRELP, MAGT1, C8B, CPN2, C4A, OLFML1, CTSG, CPA3, LUM, CMA1, FN1, PCOLCE, LYZ, SOD3, DCN, COL1A1, COL3A1, COL1A2, CFHR1, CEACAM6, CD109, OGN, COL6A3, FMOD |
| KW-0379~Hydroxylation | 8 | 5.60E-05 | COL1A1, COL3A1, COL1A2, COL14A1, PROS1, COL6A2, COL6A1, COL6A3 |
| KW-0873~Pyrrolidone carboxylic acid | 6 | 1.14E-04 | COL1A1, COL1A2, LUM, SERPINF1, FN1, FMOD |
| **Downregulated proteins: Cellular components** | | | |
| KW-0964~Secreted | 34 | 3.08E-17 | APCS, TNXB, COL14A1, PROS1, PON1, C4BPA, PLG, PRELP, C8B, CPN2, C4A, OLFML1, CTSG, LUM, CMA1, SERPINF1, FN1, APOA4, PCOLCE, PODN, LYZ, SOD3, DCN, COL1A1, MFAP4, COL3A1, COL1A2, CFHR1, COL6A2, COL6A1, SELENOP, OGN, COL6A3, FMOD |
| KW-0272~Extracellular matrix | 16 | 1.13E-14 | TNXB, COL14A1, LUM, FN1, PRELP, PODN, DCN, COL1A1, MFAP4, COL3A1, COL1A2, COL6A2, OGN, COL6A1, COL6A3, FMOD |
| KW-0345~HDL | 2 | 0.37824533 | PON1, APOA4 |
| KW-0034~Amyloid | 2 | 0.37824533 | APCS, LYZ |

**Supplementary Table S6**: MS2 spectra of six proteins among differentially expressed proteins identified with one peptide

|  | Accession | Gene symbol | Coverage (%) | Peptide match |
| --- | --- | --- | --- | --- |
| 1 | P15260 | IFNGR1 | 2 | 1 |
| 2 | Q9UPI3 | FLVCR2 | 2 | 1 |
| 3 | Q7Z739 | YTHDF3 | 2 | 1 |
| 4 | Q13232 | NME3 | 10 | 1 |
| 5 | P00846 | MT-ATP6 | 4 | 1 |

Accession # P15260 (IFNGR1)


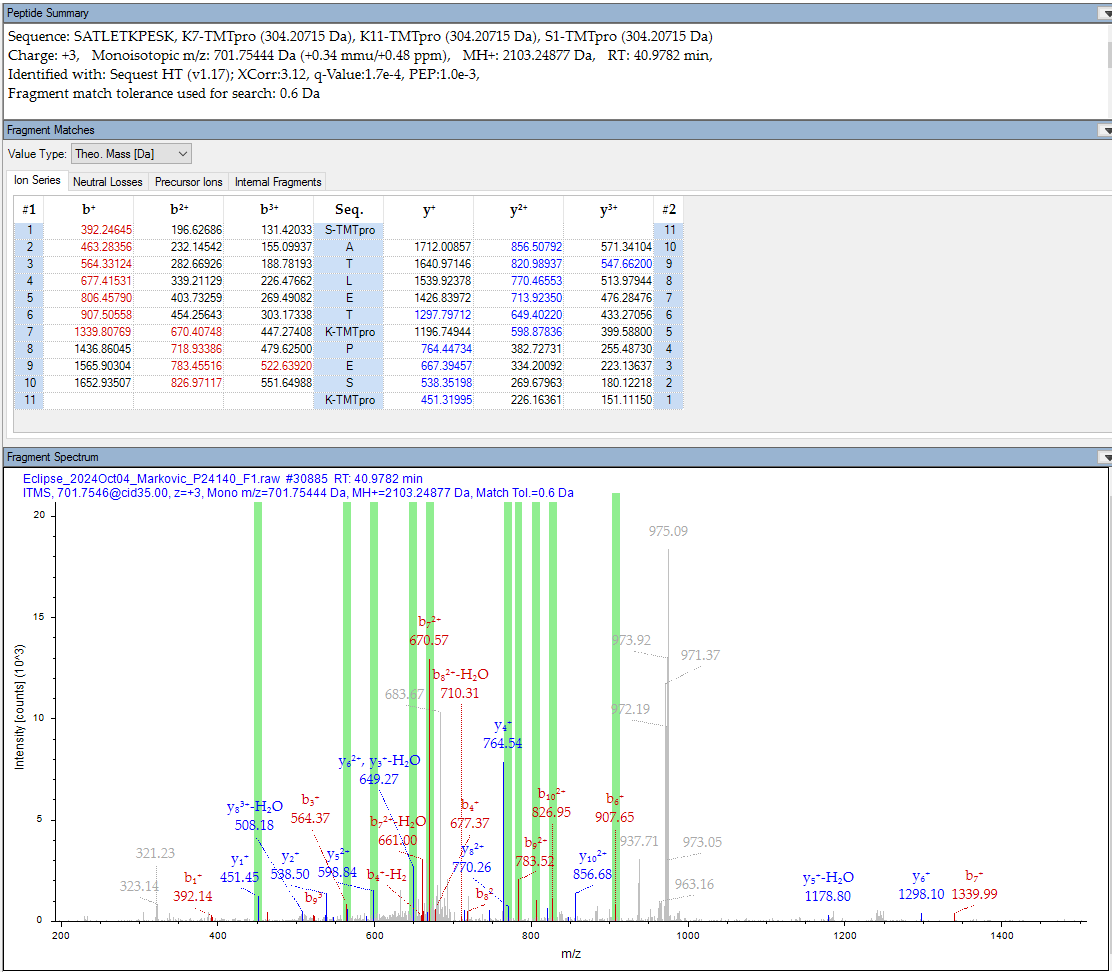


Accession # Q9UPI3 (FLVCR2)


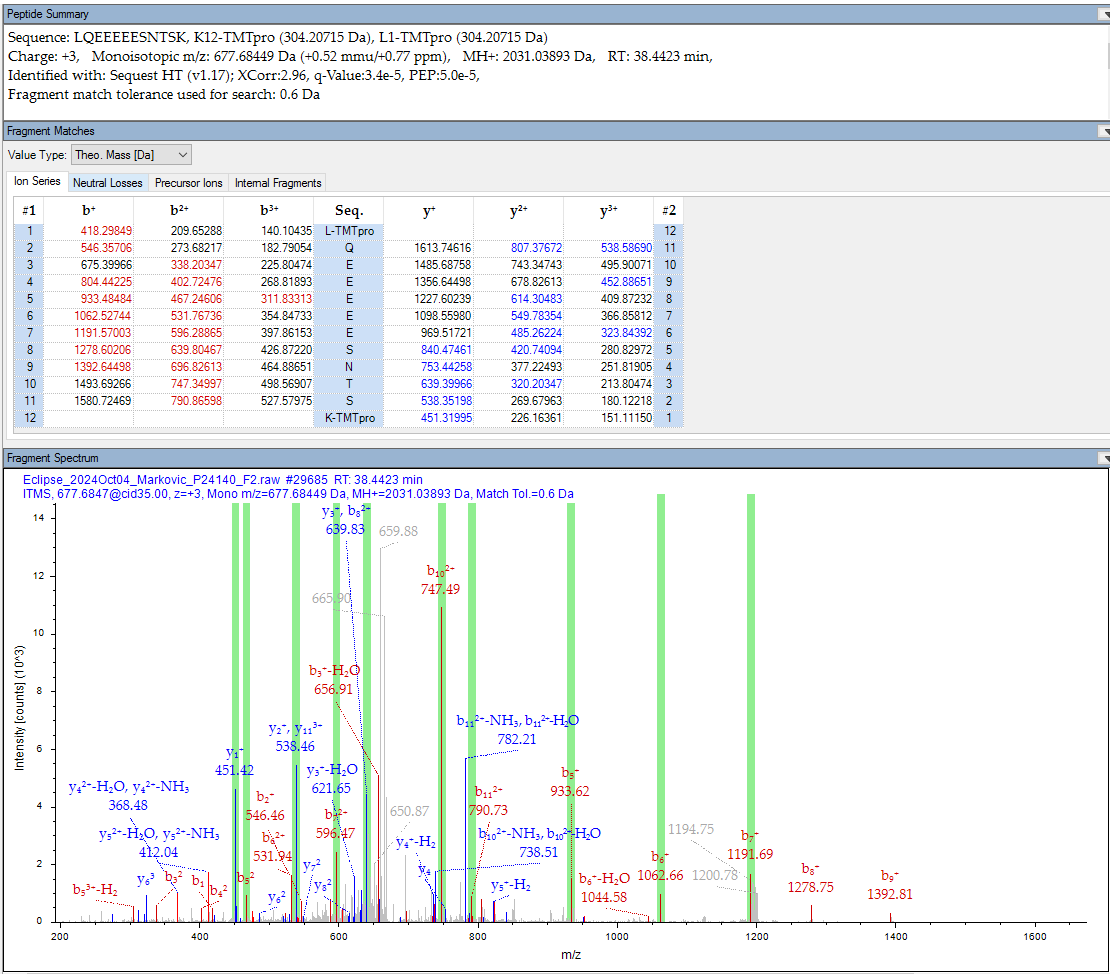


Accession # Q7Z739 (YTHDF3)


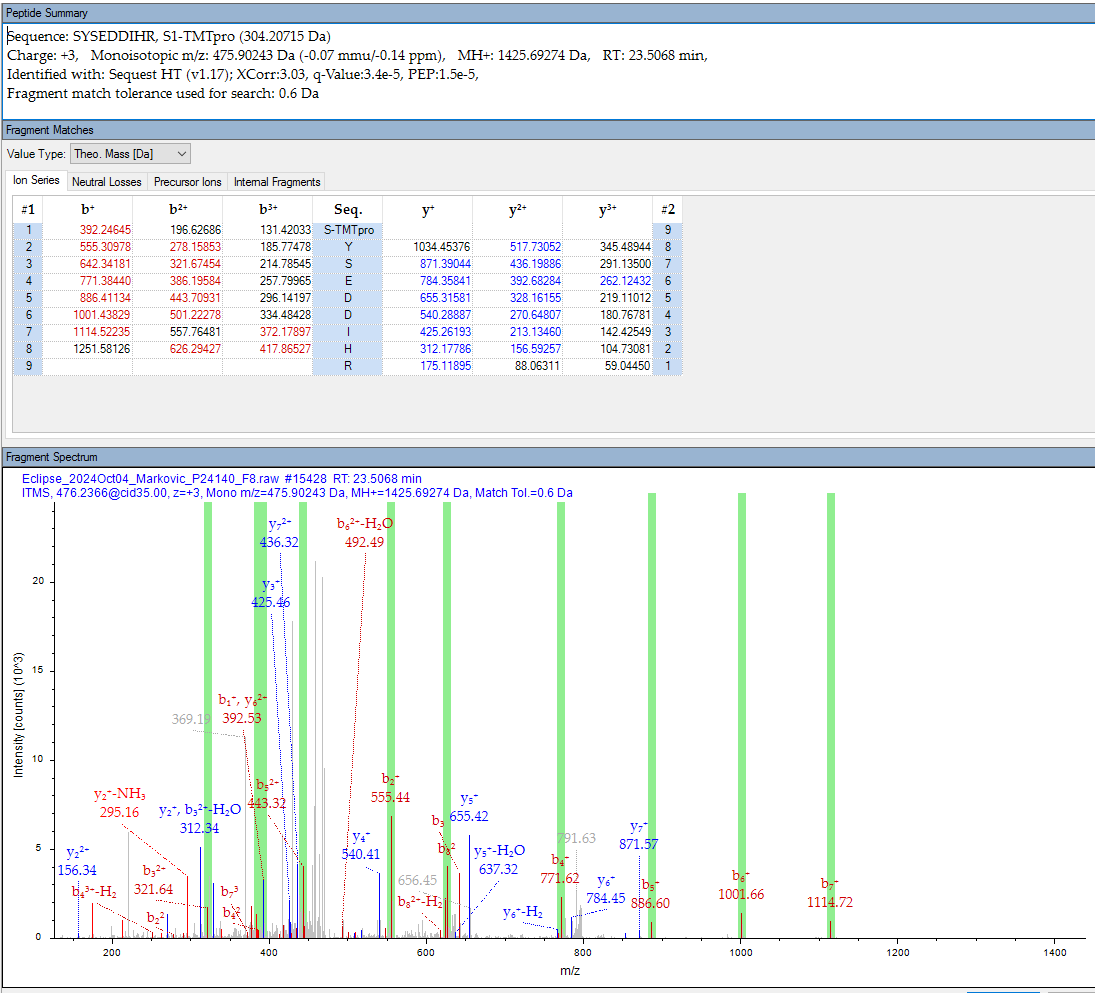


Accession # Q13232 (NME3)


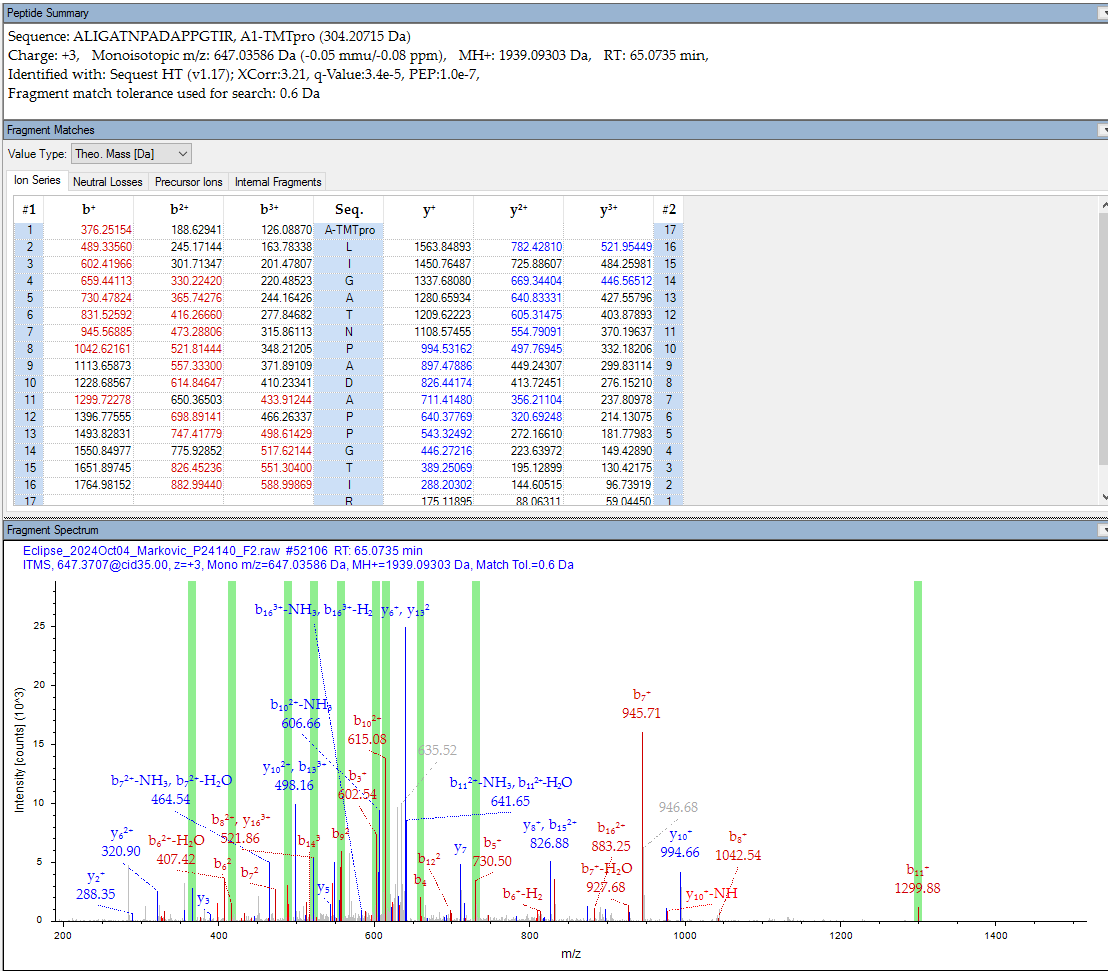


Accession # P00846 (MT-ATP6)


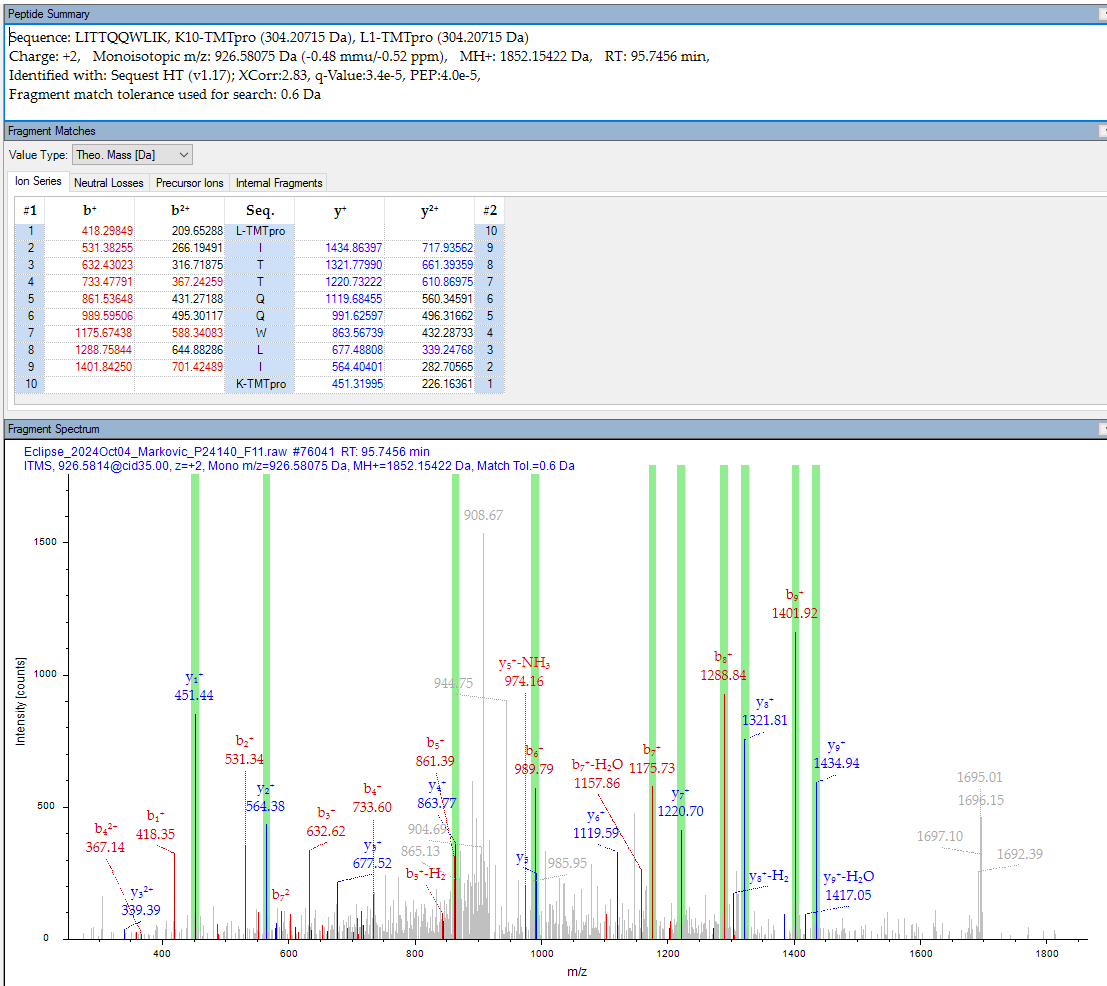

Supplement: Supplemental Materials [file mmc3.docx]
